# Pharmacological evaluation of enantiomerically separated positive allosteric modulators of cannabinoid 1 receptor, GAT591 and GAT593

**DOI:** 10.3389/fphar.2022.919605

**Published:** 2022-10-25

**Authors:** Asher L. Brandt, Sumanta Garai, Ayat Zagzoog, Dow P. Hurst, Lesley A. Stevenson, Roger G. Pertwee, Gregory H. Imler, Patricia H. Reggio, Ganesh A. Thakur, Robert B. Laprairie

**Affiliations:** ^1^ College of Pharmacy and Nutrition, University of Saskatchewan, Saskatoon, SK, Canada; ^2^ Department of Pharmaceutical Sciences, Bouvé College of Health Sciences, Boston, MA, United States; ^3^ Center for Drug Discovery, University of North Carolina Greensboro, Greensboro, NC, United States; ^4^ School of Medicine, Medical Sciences and Nutrition, Institute of Medical Sciences, University of Aberdeen, Aberdeen, Scotland, United Kingdom; ^5^ Centre for Biomolecular Science and Engineering, Naval Research Laboratory, Washington, DC, United States; ^6^ Department of Pharmacology, Faculty of Medicine, Dalhousie University, Halifax, NS, Canada

**Keywords:** type 1 cannabinoid receptor (CB1R), positive allosteric modulator, allosteric agonist, molecular pharmacology, *In silico* modeling, molecular mechanics-generalized born surface area (MMGBSA)

## Abstract

Positive allosteric modulation of the type 1 cannabinoid receptor (CB1R) has substantial potential to treat both neurological and immune disorders. To date, a few studies have evaluated the structure-activity relationship (SAR) for CB1R positive allosteric modulators (PAMs). In this study, we separated the enantiomers of the previously characterized two potent CB1R ago-PAMs GAT591 and GAT593 to determine their biochemical activity at CB1R. Separating the enantiomers showed that the *R*-enantiomers (GAT1665 and GAT1667) displayed mixed allosteric agonist-PAM activity at CB1R while the *S*-enantiomers (GAT1664 and GAT1666) showed moderate activity. Furthermore, we observed that the *R* and *S*-enantiomers had distinct binding sites on CB1R, which led to their distinct behavior both *in vitro* and *in vivo*. The *R*-enantiomers (GAT1665 and GAT1667) produced ago-PAM effects *in vitro*, and PAM effects in the *in vivo* behavioral triad, indicating that the *in vivo* activity of these ligands may occur *via* PAM rather than agonist-based mechanisms. Overall, this study provides mechanistic insight into enantiospecific interaction of 2-phenylindole class of CB1R allosteric modulators, which have shown therapeutic potential in the treatment of pain, epilepsy, glaucoma, and Huntington’s disease.

## 1 Introduction

The endogenous cannabinoid system (ECS) is composed of endogenous ligands (e.g. anandamide [AEA] and 2-arachidonoylglycerol [2-AG]), anabolic and catabolic enzymes, and receptors (the predominant receptors being the type 1 and 2 cannabinoid receptors [CB1R, CB2R]) (Di Marzo, 2018; Estrada and Contereras, 2020). The ECS is ubiquitous in the human body (Patel et al., 2021). Both CB1R and CB2R are class A G protein-coupled receptors (GPCRs) (Lutz, 2020). CB1R is most-abundant in the brain and central nervous system (CNS) whereas CB2R is most-abundant in cells of the immune system (Iannotti et al., 2016; Hryhorowicz et al., 2019). The primary role of CB1R is to regulate mood, diet, sleep and pain sensation whereas CB2R regulates immune responses (Iannotti et al., 2016). Both receptors are activated by the endogenous ligands AEA and 2-AG (Carrera et al., 2020). Due to the ubiquitous nature of CB1R and CB2R, the ECS is considered a potential target for a wide array of diseases, but CB1R in particular is considered a target for the treatment of pain and neurological disorders such as Parkinson’s disease, Huntington’s disease, and epilepsy (Perez-Olives et al., 2021).

The intoxicating effects of *Cannabis* represent a major limitation to its use as medicine. The intoxicating effects of *Cannabis* are thought to be due to ∆^9^-tetrahydrocannabinol (THC) binding to the orthosteric site of CB1R (Slivicki et al., 2020). It has previously been hypothesized that the intoxicating properties of CB1R activation could be avoided if a drug bound to the allosteric site of the receptor promoted endogenous ligand activation without direct activation of CB1R because the endogenous cannabinoids AEA and 2-AG are not known to produce intoxicating effects, tolerance, or dependence (Slivicki et al., 2020). Allosteric modulators may be positive allosteric modulators (PAMs), negative allosteric modulators (NAMs), or silent allosteric modulators (Leo and Abood, 2021). A PAM enhances the effect of the primary ligand, a NAM reduces the effect of the primary ligand, and a silent allosteric ligand does not affect the pharmacology of the orthosteric ligand (Mielnik et al., 2021). The first described CB1R allosteric modulator was the indole carboxamide Org27569, which later drove the development of indole sulfonamides as potent CB1R NAMs (Greig et al., 2016; Dopart et al., 2018).

**FIGURE 1:**
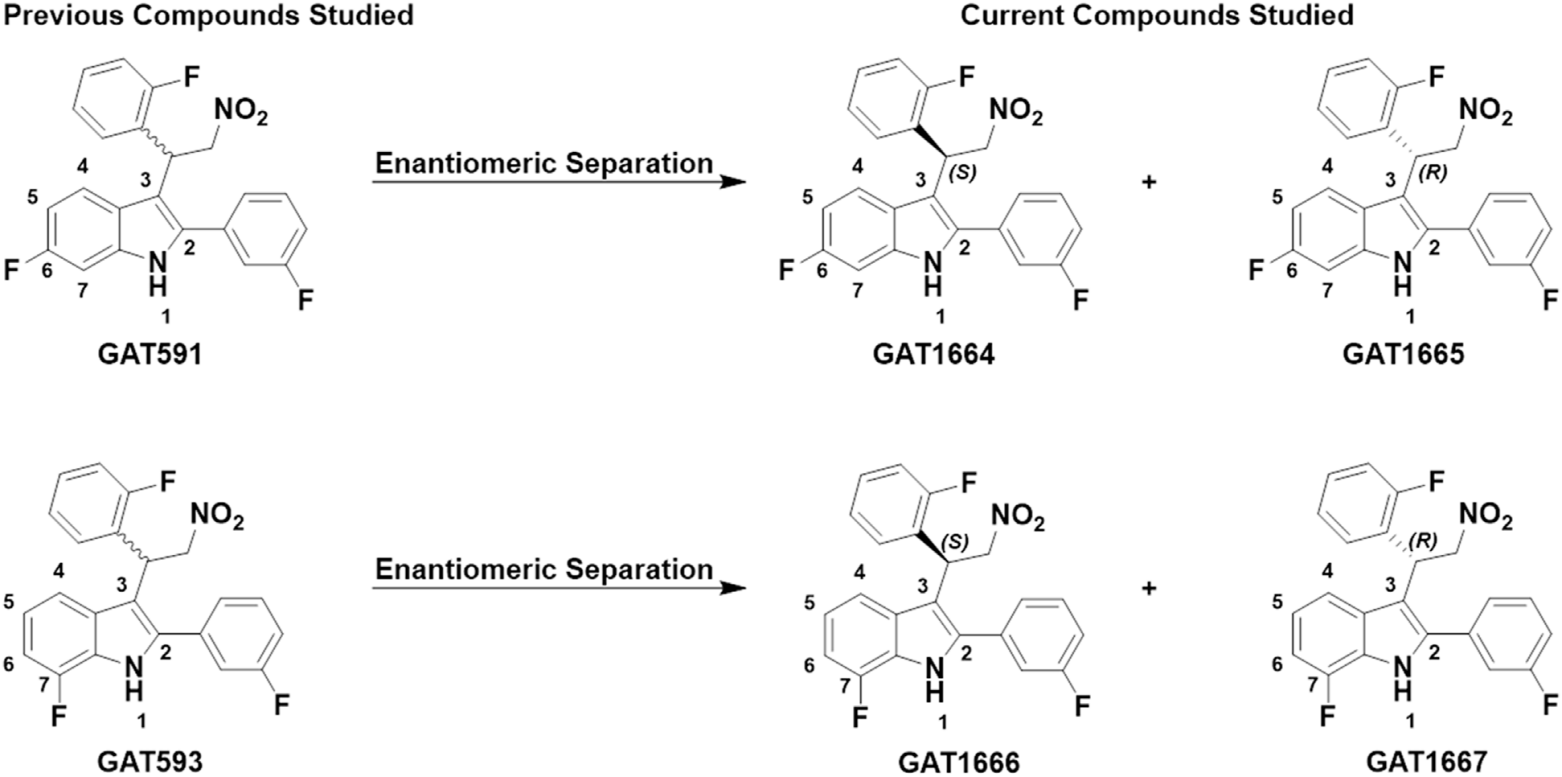
GAT591 previously studied was separated out into its respective enantiomers (GAT1664 and GAT1665) and GAT593 was separated out into its respective enantiomers (GAT1666 and GAT1667). The difference between GAT1664 and GAT1665 vs. GAT1666 and GAT1667 is the position of the fluorine in the indole ring.

In a previous paper, we reported on the pharmacology of racemic CB1R allosteric modulators GAT591 and GAT593 (Figure 1), wherein we observed these ligands have both allosteric-agonist and positive allosteric modulator (ago-PAM) properties (Garai et al., 2020). We found that GAT591 and GAT593 act at allosteric sites due to their ability to enhance the binding of [^3^H]CP55,940 at CB1R and at the same time modulate the receptor in absence of an orthostreric ligand (Garai et al., 2020). Biased agonism describes the ability of a ligand to preferentially activate one signalling pathway compared to another; for example G protein-*versus* βarrestin-mediated signaling (Leo and Abood, 2021; Patel et al., 2021). Our previous work has shown that the racemic mixtures GAT591 and GAT593 displayed bias towards Gα_i/o_ signaling as compared to βarrestin (Garai et al., 2020), which earlier work indicated was correlated with improved cell viability (Laprairie et al., 2016, 2019). Additional studies from our group support the idea that G protein bias of PAMs is correlated with improved outcomes in rodent models of Huntington’s disease, pain, and absence epilepsy (Laprairie et al., 2019; Slivicki et al., 2020; Roebuck et al., 2021); whereas βarrestin bias may be correlated with reduced cell viability and increases pathology in animal models of absence epilepsy and Huntington’s disease (Laprairie et al., 2016; Roebuck et al., 2020). One of the challenges in developing ligands for GPCRs is biased agonism (Leo and Abood, 2021; Patel et al., 2021). To further understand molecular mechanism(s) of action and probe for potential enantio specific interaction, we separated the enantiomers, determined their absolute stereochemistry and did biochemical characterization using cAMP and βarrestin2 assays to determine their allosteric-agonist and PAM activity. Previously we observed the distinct pharmacology between the enantiomers of (±)-GAT211, where (*R*)-GAT228 was an allosteric-agonist and the opposite enantiomer, (*S*)-GAT229, showed PAM activity at CB1R within the assays and cell line used (Laprairie et al., 2017). In this study we observed that the enantiomers of GAT591 and GAT593 display unique *in vitro* and *in vivo* effects that are likely to be associated with their unique modes of binding to CB1R.

## 2 Experimental section

### 2.1 *In vitro* evaluation

#### 2.1.1 Compounds used

CP55,940 and THC were purchased from Cayman (Ann Arbor, MI) and Sigma-Aldrich (Mississauga, ON), respectively. All other tested compounds were obtained from Dr. Ganesh Thakur, Northeastern University. All compounds were initially dissolved in dimethylsulfoxide (DMSO) and diluted in a 10% DMSO solution in phosphate-buffered saline (PBS). Compounds were added directly to cell culture at the time and concentrations indicated at a final concentration of 0.1% DMSO.

#### 2.1.2 Cell culture

HitHunter (cAMP) and PathHunter (βarrestin2) Chinese hamster ovary (CHO)-K1 cells stably-expressing human CB1R (hCB1R) from DiscoveRx (Eurofins, Freemont, CA) were maintained at 37°C, 5% CO_2_ in DMEM/F-12 containing 10% fetal bovine serum (FBS) and 5 U × 10^4^ U penicillin/streptomycin (ThermoFisher, Mississauga, ON). In addition, 800 μg/ml geneticin was used for CHO-K1 hCB1R HitHunter cAMP cells and 800 μg/ml geneticin and 300 μg/ml hygromycin B was used for CHO-K1 hCB1R PathHunter βarrestin2 cells.

#### 2.1.3 HitHunter cAMP assay

cAMP inhibition was performed in the presence of 10 µM forskolin (FSK) using the DiscoveRx HitHunter assay in hCB1R CHO-K1 cells. Cells (16,000 cells/well in 96 well plates) were incubated overnight in Opti-MEM (Invitrogen) containing 1% FBS at 37°C and 5% CO_2_. Opti-MEM media was removed and replaced with cell assay buffer (DiscoveRx) and then cells were simultaneously treated with 10 µM FSK and experimental compounds (0.10 nM–10 µM) for 90 min cAMP antibody solution and working detection solutions were added according to the manufacturer’s protocol (DiscoveRx), and cells were incubated for 60 min at room temperature. cAMP solution A was added according to the manufacturer’s protocol (DiscoveRx) and cells were incubated for an additional 60 min at room temperature before chemiluminescence was measured on a Cytation5 plate reader (top read, gain 200, integration time 10 s). Data are presented as percent maximal CP55,940-dependent inhibition of cAMP accumulation.

#### 2.1.4 PathHunter βarrestin2 assay

βarrestin2 recruitment was determined using the hCB1R CHO-K1 cell PathHunter assay (DiscoveRx). Cells (16,000 cells/well in 96 well plates) were incubated overnight in Opti-MEM (Invitrogen) containing 1% FBS at 37°C and 5% CO_2_. Cells were then simultaneously treated with experimental compounds (0.10 nM–10 µM) for 90 min. Detection solution was added to cells according to the manufacturer’s protocol (DiscoveRx), and cells were incubated for 60 min at room temperature. Chemiluminescence was measured on a Cytation5 plate reader (top read, gain 200, integration time 10 s). Data are presented as percent maximal CP55,940-dependent stimulation.

#### 2.1.5 [^3^H]CP55,940 radioligand displacement assay

Previous work from our group has shown that CB1R PAMs enhance orthosteric agonist binding (e.g. CP55,940) (Laprairie et al., 2017; Garai et al., 2020). Radioligand binding assays were carried out with 1 nM [^3^H]CP55,940 in Tris buffer (75 mM Tris-HCl, 12.5 mM MgCl_2_, 1 mM ethylenediaminetetraacetic acid (EDTA), 1% bovine serum albumin (BSA), pH 7.4) with a total assay volume of 200 µl. The assay began with the addition of transfected hCB1R CHO-K1 cell membranes (25 µg protein per well). The assays were left to equilibrate at room temperature for 2 h before vacuum filtration using a Millipore Sigma 12-well sampling manifold and filter paper that had been soaked in wash buffer. Each reaction well was washed 3 times with a 2 ml aliquot of Tris-binding buffer. The filters were removed then submerged in 5 ml of scintillation fluid (Ultima Gold F, PerkinElmer, Buckinghamshire, United Kingdom). Radioactivity was quantified by liquid scintillation spectrometry. Specific binding was defined as the difference between binding that occurred in the presence and in the absence of 1 µM unlabeled CP55,940. Data are presented as percent [^3^H]CP55,940 bound.

#### 2.1.6 [^35^S]GTPγS assay

This assay was conducted as described in previous reports (Garai et al., 2020). To summarize, the assay was completed in the presence of [35S]GTPγS (0.1 nM), GDP (30 µM), GTPγS (30 µM) using membranes derived from CHO-K1 cells (1 mg/ml) overexpressing hCB1R. Assay buffer consisted of 50 mM Tris, 10 mM MgCl2, 100 mM NaCl, 0.2 mM EDTA and 1 mM dithiothreitol (DTT) at pH 7.4. Membranes were incubated at 30°C for 90 min in a total volume of 500 ml. Reactions were ended by the addition of ice-cold wash buffer (50 mM Tris and 1 mg/ml BSA, pH 7.4) followed by rapid filtration under vacuum through pre-soaked Whatman GF/B glass-fibre filters in a 24-well sampling manifold (Brandel Cell Harvester; Brandel Inc., Gaithersburg, MD, United States). Reaction wells were washed six times with a 1.2 ml aliquots of Tris-binding buffer. Filters were subsequently oven-dried for 60 min and placed in 3 ml of scintillation fluid (Ultima Gold XR, PerkinElmer). Bound radioactivity was determined by liquid scintillation counting. Basal [35S]GTPγS binding was determined in the presence of 20 mM GDP without any compounds present. Non-specific binding was determined in the presence of 10 mM GTPγS.

### 2.2 *In vivo* evaluation

#### 2.2.1 Triad assessment in mice

Male C57BL/6 mice (Charles River, Senneville, QC) between 4 and 7 months of age were used for these studies. Animals were group housed at the Laboratory Animal Services Unit (LASU) at the University of Saskatchewan (3-5 animals/cage) with a standard 12:12 light-dark cycle, *ad libitum* access to food and water, and environmental enrichment. Compounds administered intraperitoneally (i.p.) were prepared in vehicle [ethanol and cremophor in saline (1:1:8)]. Catalepsy was assessed in the bar holding assay 5 min after drug administration. Mice were placed so that their forepaws clasped a 0.7 cm ring clamp positioned 4.5 cm above the surface of the testing space (Zagzoog et al., 2021). The length of time the ring was held was recorded in seconds. The trial ended if the mouse turned its head or body or made 3 consecutive escape attempts to a maximum of 60 s. Body temperature was measured by rectal thermometer 15 min after drug administration. Anti-nociceptive effects were measured in the warm water (52 ± 2°C) tail-flick test 20 min after drug administration. Response in this case was defined by the removal of the tail from the warm water, with a maximal response time of 20 s. Catalepsy and tail flick data are presented as percent maximum possible effect (MPE). Compounds were administered at the doses indicated. Experimenters were blinded to treatment for all behavioral assessments and analyses. Animals were purchased, rather than bred, to reduce animal numbers. In all cases, experiments were performed with the approval of the University Animal Care Committee (UACC) at the University of Saskatchewan and are in keeping with the guidelines of the Canadian Council on Animal Care (CCAC) and the ARRIVE guidelines (Kilkenny et al., 2010).

### 2.3 *In silico* evaluation

#### 2.3.1 Ligand Preparation

A conformational analysis was performed on GAT1664, GAT1665, GAT1666, and GAT1667 using Spartan’18 V1.4.5 (Spartan, 2018). A conformational search of each compound was run at ground state with molecular mechanics force fields (MMFF) (Spartan, 2018). Semi empirical calculations utilizing the PM3 Hamiltonian were carried out to calculate the equilibrium geometry of each conformer. After duplicates were eliminated, HF/6-31G* was applied to the remaining rotamers to obtain the global minimum energy conformation (Spartan, 2018).

#### 2.3.2 CB1R model

The CB1R model has been described in detail in Hurst et al. (2019), but in brief: The CB1R model used in this study was based on the CB1R activated state crystal structure (PDB-ID: 5XRA) (Hua et al., 2017) and the cryo-EM CB1/Gi bound structure (PDB-ID: 6N4B) (Krishna Kumar et al., 2019). Structures were prepared with the Protein Preparation protocol (Suite 2019-1, Schrödinger, Inc.), mutations in the 5XRA structure were returned to wild-type, and both models were inspected for close contacts and crystal packing effects. Modification of the 5XRA structure to create the CB1R model involved calculating low free energy conformations for TMH2/7 to accommodate mutation data for S7.39, F2.61, F2.64 (Kapur et al., 2007; Shim et al., 2011). In addition, because of loop compression in the 5XRA structure, the IC1 loop from the 6N4B Gi bound structure was used in the model. TMH4 in the 5XRA CB1R* structure has tight crystal contacts on its IC end with TMH1 of another bundle because of antiparallel packing. For this reason, the conformation of TMH4 in the 6N4B Gi bound structure was used in the model. Modeller was used to remodel the EC3 loop (D6.58 to T7.33) and allow K (373) to interact with D2.63, consistent with mutation results (Fiser and Sali, 2003; Marcu et al., 2013). Modeller was also used to extend and model the N-terminus to S (88) with inclusion of the C (98) to C (107) disulfide bridge (Fay and Farrens, 2013, 2015). The receptor/ligand complex was energy minimized in Prime (Suite 2019-1, Schrödinger, Inc.). The Prime implicit membrane functionality was employed. Hydrophilic residues facing the binding crevice and within the low dielectric region of the implicit membrane were excluded from the low dielectric *via* exclusion spheres placed on each residue. The Generalized Born/Surface Area (GB/SA) continuum solvation model for water was used with the dielectric set to 80 outside of the implicit membrane region and 2 within. A truncated Newton conjugate gradient minimization was performed using the OPLS3e force field for one iteration up to a maximum of 1,000 steps and with a 0.1 kcal/mol gradient endpoint. Constraints of 1 kcal/mol were placed on the C-alpha atoms of residues R3.50, Y5.58, L6.33, and Y7.53 to prevent the intracellular opening present in the R* structures from closing during the minimization.

#### 2.3.3 Binding Site Identification

To identify potential binding site(s) for GAT1664, GAT1665, GAT1666 and GAT1667 at CB1R, we used the Forced-Biased Metropolis Monte Carlo simulated annealing program (MMC) as the first step (Guarnieri and Mezei, 1996; Clark et al., 2006). The MMC method has been used successfully to identify water binding sites in proteins and on DNA to identify potent and novel p38 kinase inhibitors; to identify thermolysin and T4 and lysosome biding sites; and, to identify the binding site of the negative allosteric modulator, pregnenolone (Morales et al., 2016; Zhang et al., 2020). In MMC, the molecule of interest is first divided into smaller fragments. A series of grand canonical ensembles of a molecular fragment interacting with the protein in a large simulation box are created. The Chemical potential of the system is then annealed at descending chemical potential levels, with each new level starting from the last ensemble generated from the previous one. At each step, fragment ligand poses are sampled throughout the box and over the entire protein. Fragments are treated as rigid solvents that are inserted and deleted millions of times until the lowest energy configuration is found at the explored annealing level. As the chemical potential is annealed, the number of fragments in the box decreases because the method eliminates any fragment that has a poorer free energy of interaction than the annealing level. The output of the calculation is an ensemble of ligand poses at each chemical potential level in the annealing schedule. The method is repeated for each molecular fragment. Data analysis is performed using the GENS tool in the MMC program available from the Mihaly Mezei Laboratory (Mezei, 2011).

Of particular interest in MMC calculations are fragments that persist at particular sites on the protein throughout the annealing schedule, since these fragments clearly have high affinity for those sites. Receptor regions at which all molecular fragments of a studied ligand collect are identified from MMC output. This set of sites is then refined to include only those sites at which the order of the fragments reflects the structure of the entire molecule.

GAT1664 and GAT1665 were broken into fragments that included a 6-fluoro-indole ring, a 2-fluoro-phenyl ring and a 3-fluoro-phenyl ring. GAT1666 and GAT1667 were broken into fragments that included 7-fluoro-indole ring, 2-fluoro-phenyl ring and 3-fluoro-phenyl ring. All fragments were prepared with partial charges using the Amber 2002 force field, a point-charge force field for molecular mechanics simulations of proteins based on condensed-phase quantum MMC (Wang et al., 2018). Six MMC runs were performed in which our CB1R receptor model was immersed in a box filled with copies of one of these fragments. Analysis of the MMC runs for GAT1664 and GAT1666 revealed that while each fragment bound to multiple positions on the CB1R receptor, there was only one region in which all fragments clustered. This was at the extracellular end of TMH2/3, just beneath the EC1 loop. Y2.59, a polar residue that faces lipid was found to attract the nitro group fragment, while D2.63 consistently was found interacting with the fluorinated indole fragments.

*Docking*: Once the two sites were confirmed, the structures of GAT1664-GAT1667 were docked in the intracellular TMH1/2/4 site with H2.41, F4.46, and W4.50 used as direct interaction sites. GAT1664 and GAT1666 were docked in the TM2/3/EC1 site with Y2.59 and D2.63 as direct interaction sites. In addition, after extensive previously published molecular dynamics calculations on unfluorinated versions of the GAT compounds (GAT1600-3), R (148) was modeled to interact directly with the carbonyl oxygens of the last turn of TMH1 and not the nitro group within the compounds (Garai et al., 2021). The receptor/ligand complexes were energy minimized in Prime (Suite 2019-1, Schrödinger, Inc.). The Prime implicit membrane functionality was employed. Hydrophilic residues facing the binding crevice and within the low dielectric region of the implicit membrane were excluded from the low dielectric *via* exclusion spheres placed on each residue. The Generalized Born/Surface Area (GB/SA) continuum solvation model for water was used with the dielectric set to 80 outside of the implicit membrane region and 2 within. A truncated Newton conjugate gradient minimization was performed using the OPLS3e force field for 1 iteration, up to a maximum of 1,000 steps and with a 0.1 kcal/mol gradient endpoint. Constraints of 1 kcal/mol placed on the c-alpha atoms of residues R3.50, Y5.58, L6.33, and Y7.53 were set to prevent the intracellular opening from closing during the minimization. The resulting docks were refined with the Induced Fit protocol (Suite 2019-1, Schrödinger, Inc.). The Glide box size was set to 12 Å3 centered on the ligand and the SP docking algorithm employed. Residues within 5 Å of the docked ligand were included in the Prime refinement stage, except in the case of the TM2/3 PAM site where S2.60 and K3.28 were excluded based on mutation data (Song and Bonner, 1996; Kapur et al., 2007). The implicit membrane previously used during the initial Prime minimization was employed here as well.

#### 2.3.4 MMGBSA analysis

Each of the GAT1664-GAT1667 ligand-receptor complexes were evaluated *via* MMGBSA. This was the scoring function used to evaluate each complex and not Glide scores. This method is used to estimate the relative binding affinities for a list of ligands (reported in kcal/mol). As the MMGBSA binding energies are approximate free energies of binding, a more negative value indicated stronger binding. For this computation, the VSGB solvation model was employed while the chosen force field OPLS4 was used.

### 2.4 Statistical analysis

Data related to EC_50_ and *E*_max_ in Figures 3, 4 were obtained with nonlinear regression models (3-parameter model, GraphPad Prism 9.0, San Diego, CA). Results were calculated as percent response relative to the reference agonist CP55,940 (Figure 3), percent [^3^H]CP55,940 bound (Figure 4A), or percent stimulation above baseline (Figures 4B,C). The 3-parameter model was used for [^3^H]CP55,940 binding assays (Figure 4A) because standard radioligand competition models that fit for *K*_d_ do not appropriately account for allosteric interactions. Significance was determined by one- or two-way ANOVA followed by Tukey’s or Bonferroni’s post-hoc test as indicated. *p* < 0.05 was considered significant and *p* values were only employed where ANOVA was used. Data related to Figure 5 was analyzed in GraphPad Prism 9.0 was used to analyze *in vivo* data and *p* < 0.05 was considered to be statistically significant. Data are expressed as mean ± SEM. Group sizes for all experiments are described in figure legends.

## 3 Results and discussion

Our *in vitro* study explored CB1R-dependent modulation of cAMP inhibition and βarrestin2 recruitment in CHO-K1 cells stably-expressing hCB1R with or without CP55,940. Inhibition of cAMP accumulation is Gα_i/o_-protein mediated whereas recruitment of βarrestin2 is G-protein independent (Hryhorowicz et al., 2019). In a previous paper, GAT591 and GAT593 displayed mixed agonist and PAM (i.e. ago-PAM) activity (Garai et al., 2020). The compounds GAT591 and GAT593 contain a 50:50 racemic mixture of both *R* and *S* enantiomers. In this paper each enantiomer was investigated to determine their allosteric agonist and PAM activity, as we previously observed these properties to be enantiomerically distinct in the compounds GAT211, GAT228, and GAT229 within those experimental conditions (Laprairie et al., 2017). Separating GAT591 and GAT593 into their respective enantiomers gave GAT1664, GAT1665, GAT1666 and GAT1667 (Figure 1).

### 3.1 Synthesis, chiral separation and absolute stereochemistry determination

**FIGURE 2:**
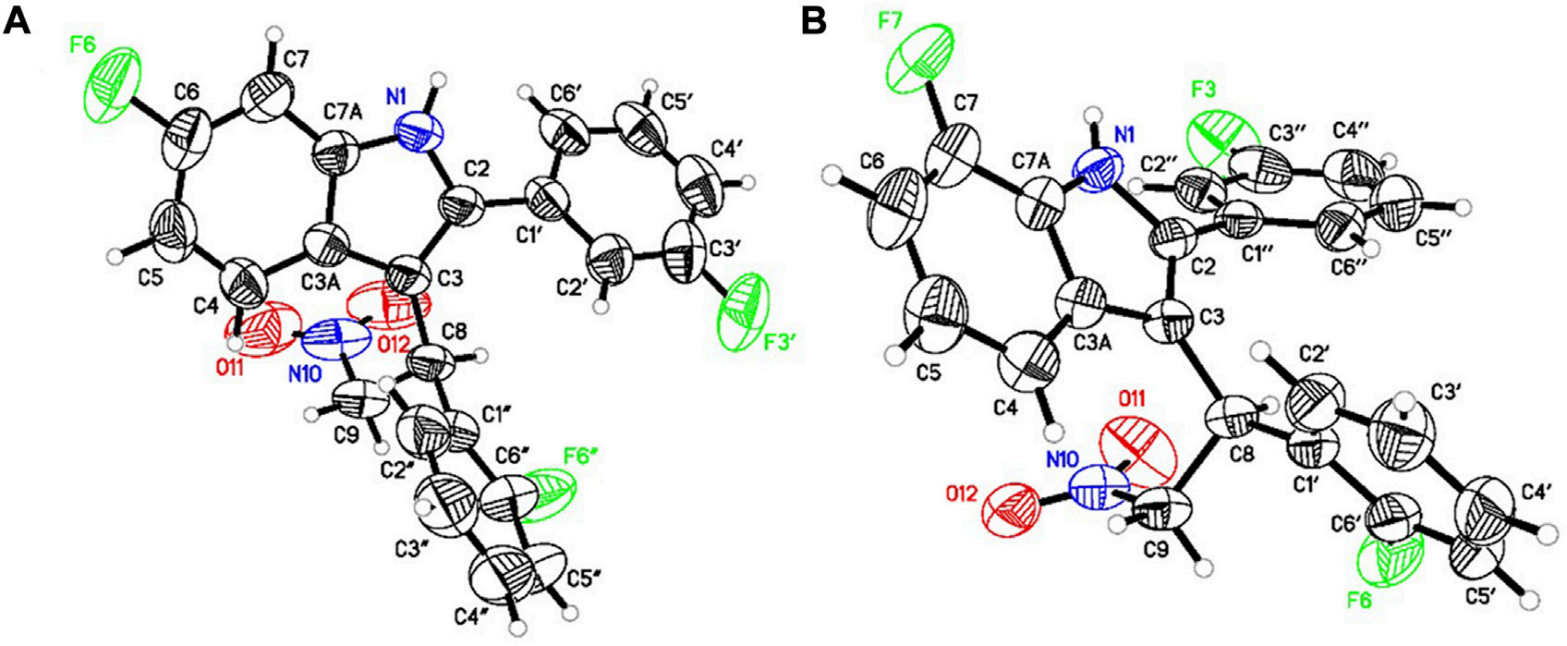
ORTEP diagram of (+)-GAT1664 [**(A)**, CCDC no. 2086717] and (+)-GAT1666 [**(B)**, CCDC no. 2086718].

We synthesized GAT591 and GAT593 on a multigram scale using our previously published method (Garai et al., 2020). Both enantiomers of each compound were separated in high optical purity (>99%) using superfluid chiral high performance liquid chromatography (HPLC). The absolute stereochemistry of (+)-GAT1664 and (+)-GAT1666 was determined by single-crystal X-ray diffraction technique (for details see supporting formation) and was found to be “*S*” for both of these enantiomers (Figure 2). Based on this study, we can predict the absolute stereochemistry of each opposite enantiomers, (-)-GAT1665 and (-)-GAT1667 as “*R*” (Figures 1, 2).

### 3.2 *In vitro* evaluation

**FIGURE 3:**
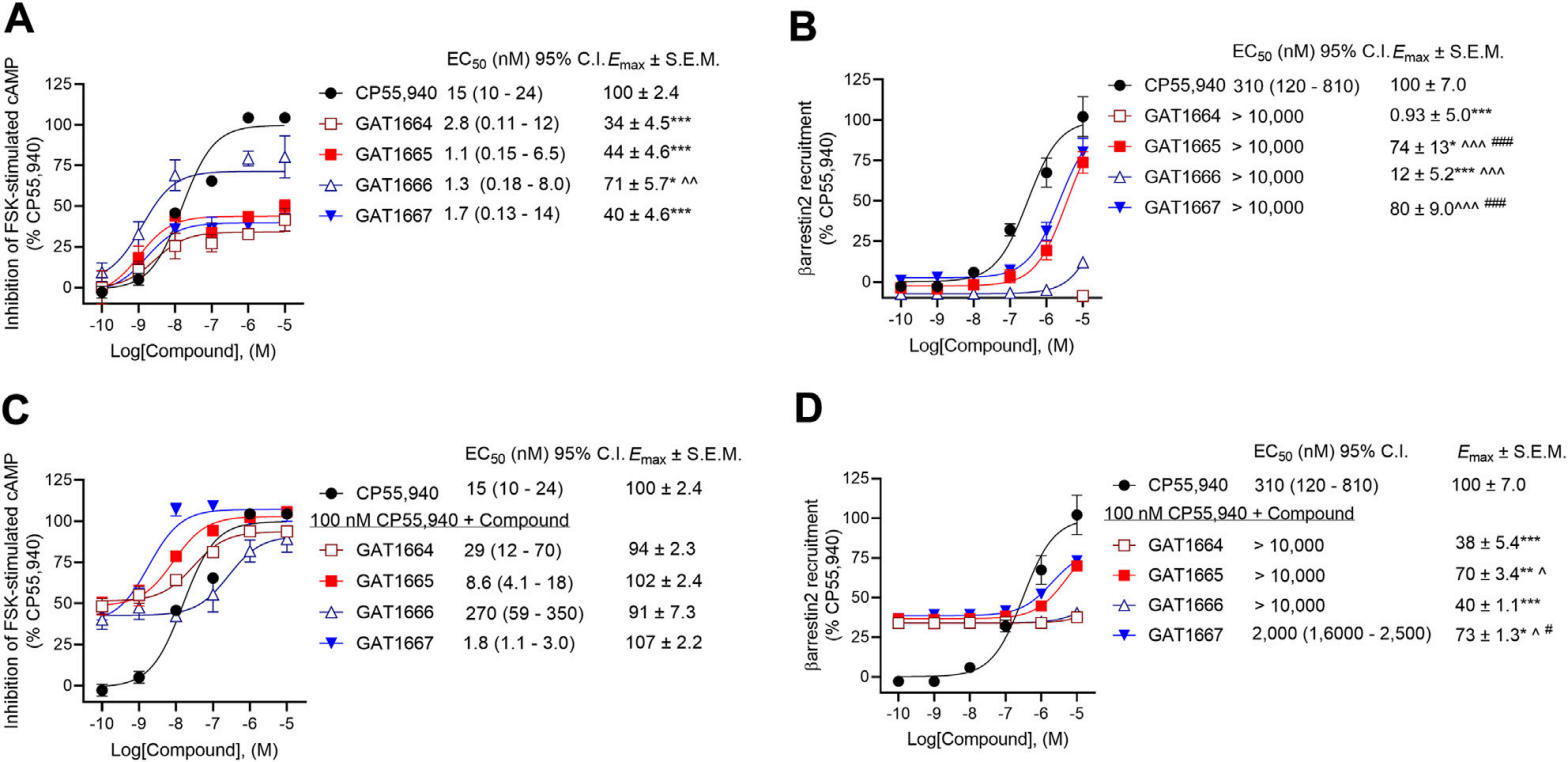
Assessment of GAT1664, GAT1665. GAT1666, and GAT1667 signaling. hCB1R CHO-K1 were treated with 0.10 nM–10 µM GAT compounds ±100 nM CP55,940. **(A)** cAMP inhibition of GAT compounds as agonists, **(B)** βarrestin2 recruitment of GAT compounds as agonists, **(C)** cAMP inhibition of GAT compounds as PAMs in the presence of 100 nM CP55,940, and **(D)** βarrestin2 recruitment of GAT compounds as PAMs in the presence of 100 nM CP55,940. cAMP inhibition and βarrestin2 recruitment data are expressed as % CP55,940 max. Data were fit to a nonlinear regression (3-parameter model, GraphPad v. 9.0) to determine EC_50_ and *E*_max_. Data are mean ± SEM (*E*_max_) or 95% CI (EC_50_); *n* = 4 independent experiments performed in triplicate, except for CP55,940 where *n* = 12 independent experiments performed in triplicate as this compound was included in all assays as a reference compound. *p < 0.05, **p < 0.01, ***p < 0.001 compared to CP55,940; ^*p* < 0.05, ^^*p* < 0.01, ^^^*p* < 0.001 compared to GAT1664, ^#^ *p* < 0.05, ^###^ *p* < 0.001 compared to GAT1666 as determined by one-way ANOVA followed by Tukey’s post-hoc test.

Previously, the racemic compounds GAT591 and GAT593 were characterized for their PAM activity in the presence of 100 nM CP55,940 (Garai et al., 2020). Here, CP55,940 displayed EC_50_ values of 15 nM and 310 nM in the cAMP inhibition and βarrestin2 recruitment assays respectively (Figure 3). Therefore, 100 nM CP55,940 was used in PAM assays in the current study for consistency between studies and as an intermediate concentration.

All enantiomers displayed G protein-mediated agonist activity at hCB1R as shown by their ability to inhibit cAMP production in the absence of an orthosteric agonist (Figure 3A). The ability of these compounds to augment orthosteric agonist signaling was tested in the presence of 100 nM CP55,940 because we have used this concentration of CP55,940 in previous studies (Garai et al., 2020) and because 100 nM CP55,940 produce an ∼65% response relative to the maximum response observed prior to fitting the data to a nonlinear regression (Figure 3). All 4 compounds increased CP55,940’s activity above that observed at the 100 nM level, consistent with either positive allosteric modulation or additive non-competitive agonism (i.e. ago-PAMs) of hCB1R in the presence of the orthosteric agonist CP55,940 in the cAMP assay (Figure 3C). As an agonist, GAT1666 displayed greater efficacy than GAT1664, and GAT1667 displayed the greatest efficacy at inhibiting cAMP as a PAM, although this was not statistically different from other compounds tested. All enantiomers displayed low nanomolar potency as agonists (Figure 3A). Although not statistically significant differences, both *R-*enantiomers (GAT1665 and GAT1667) displayed greater PAM potency and efficacy than the *S-*enantiomers (GAT1664 and GAT1666), (Figure 3C). Initial studies with the parent compound scaffold, GAT211, found that PAM activity was associated with GAT229 whereas allosteric agonist activity was associated with GAT228 (Laprairie et al., 2017). Importantly, repeated testing of GAT229 and its structural analogs over the years has shown assay-specific variability and that these enantiomers can display some allosteric agonist activity (Garai et al., 2020), including in the present study. Pure PAM activity has been observed for GAT229 in the autaptic hippocampal neuron *ex vivo* model systems (Mitjavila et al., 2018). These observations are in keeping with our earlier findings with the parent racemic ligands because PAM activity is predominantly attributable to GAT229, which shares the same spatial orientation as GAT1665 and GAT1667, and agonist activity is predominantly attributable to GAT228, which shares the same spatial orientation as GAT1664 and GAT1666 (Laprairie et al., 2017).

For consistency with cAMP inhibition experiments and previous studies (Garai et al., 2020), compounds were tested for their ability to augment CP55,940-dependent βarrestin2 recruitment with 100 nM CP55,940. Both GAT1665 and GAT1667 recruited βarrestin2 as low-potency agonists and PAMs at hCB1R (Figures 3B,D). GAT1664 did not recruit βarrestin2 to hCB1R while GAT1666 displayed a weak ability to do so as an agonist (Figures 3B,D). This suggests that as agonists, GAT1665 and GAT1667 (the *R*-enantiomers) recruit βarrestin2.

The operational model described by Kenakin et al. (2012) cannot be used to describe bias for non-competitive interactions accurately. Therefore, bias (∆∆logR) cannot be directly estimated between cAMP inhibition and βarrestin2 recruitment. Examining the potency and efficacy of compounds directly, however, we observed that as an agonist, GAT1664 displayed no activity βarrestin2 recruitment and this compound appears to heavily favour G protein-dependent inhibition of cAMP (Figure 3). GAT1666 displayed greater potency in the cAMP inhibition assay (Figure 3). GAT1665’s potency and efficacy generally favored cAMP inhibition (Figure 3). GAT1667 did appear to favour inhibition of cAMP as an agonist (Figure 3). As PAMs, GAT1664 and GAT1666 displayed no activity in the βarrestin2 recruitment assay (Figure 3). GAT1665 and GAT1667 favoured cAMP inhibition relative to βarrestin2 recruitment (Figure 3F). The parent compound GAT229 shares the same spatial orientation as GAT1665 and GAT1667 and also displayed greater potency in cAMP inhibition assays when tested as a PAM (Laprairie et al., 2017). Therefore, these data confirm previous observations that this scaffold’s spatial orientation promotes G protein PAM activity and extends earlier reports to demonstrate increased potency and efficacy of fluorine-substituted ligands as compared to parent compounds (Laprairie et al., 2017).

**FIGURE 4:**
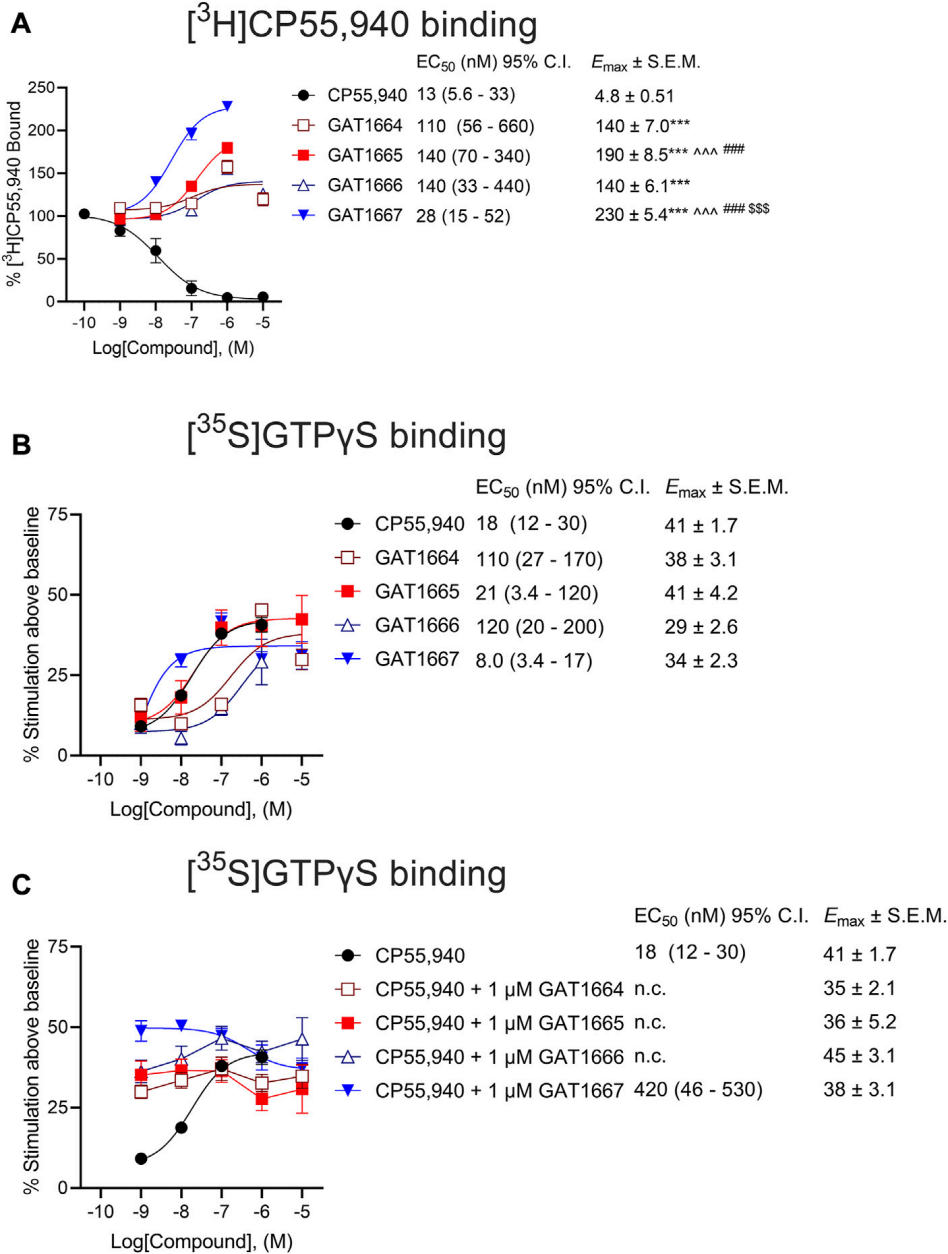
**(A)** Radioligand binding of 1 nM [^3^H]CP55,940 and **(B,C)** G protein binding of [^35^S]GTPγS to membranes from hCB1R CHO-K1 cells. hCB1R CHO-K1 cells were treated with 0.10 nM–10 µM compounds in the presence 1 nM [^3^H]CP55,940 **(A)** or 1 nM [^35^S]GTPγS **(B)**. Data are expressed as % radioligand bound **(A)** or % stimulation above baseline (i.e. vehicle) levels **(B,C)**. Data were fit to a nonlinear regression (3-parameter model, GraphPad v. 9.0) to determine EC_50_, and *E*_max_. Data are mean ± SEM (*E*_max_) or 95% CI (EC_50_); *n* = 6 independent experiments performed in triplicate, except for CP55,940 where *n* = 12 independent experiments performed in triplicate as this compound was included in all assays as a reference compound. ***p < 0.001 compared to CP55,940, ^^^*p* < 0.001 compared to GAT1664, ^$$$^ *p* < 0.01 compared to GAT1665, ^###^ *p* < 0.01 compared to GAT1666, as determined by one-way ANOVA followed by Tukey’s post-hoc test.

Next, we sought to assess the ability of these compounds to modulate orthosteric agonist binding to hCB1R. All enantiomers studied augmented [^3^H]CP55,940 binding, consistent with their proposed PAM activity, with the *R-*enantiomers GAT1665 and to a greater extent GAT1667 increasing [^3^H]CP55,940 binding (Figure 4A). GAT1667 displayed the greatest potency among the enantiomers tested (EC_50_ = 28 [15–52] nM). Enantiomers were also tested for their ability to promote G protein coupling in the GTPγS assay (Figures 4B,C). All of the enantiomers tested increased G protein coupling alone (i.e. in the absence of the orthosteric agonist CP55,940) with the *R-*enantiomers GAT1665 and GAT1667 being more potent than the *S*-enantiomers and GAT1667 in particular displaying the greatest potency (EC_50_ = 8.0 [3.4–17] nM) (Figure 4B). When 1 μM of each enantiomer was tested in the presence of CP55,940, each enantiomer increased G protein coupling which may be the result of allosteric non-competitive agonism or PAM activity (Figure 4C). Therefore, all enantiomers tested were able to stimulate G protein coupling alone or in the presence of CP55,940, which is consistent with their activity as ago-PAMs. Among the enantiomers tested, GAT1667 consistently displayed the greatest potency and efficacy.

### 3.3 *In vivo* evaluation

**FIGURE 5:**
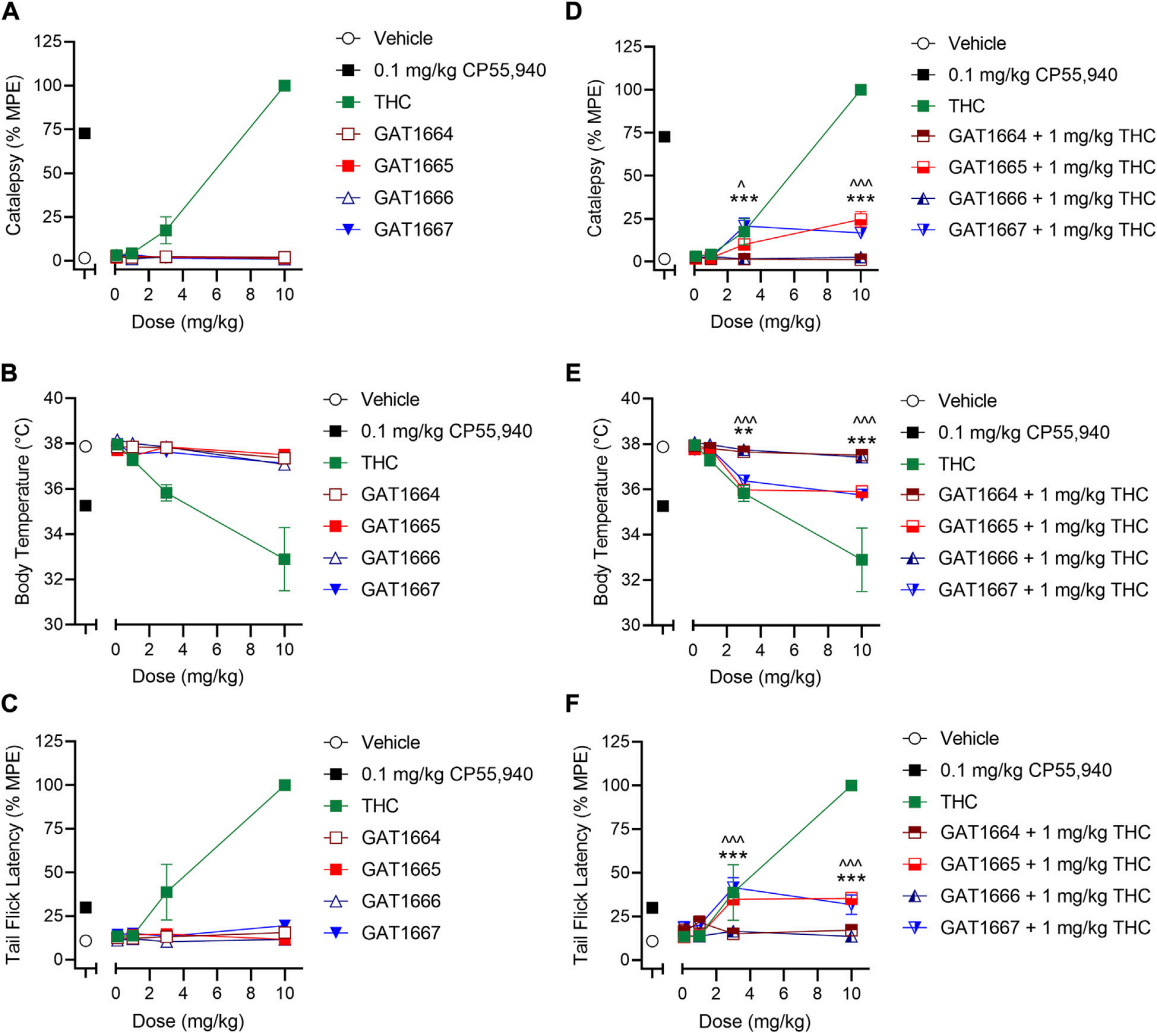
*In vivo* effects of GAT1664, GAT1665, GAT1666 and GAT1667 in male C57BL/6 mice. C57BL/6 mice were administered compounds either alone or co-administered with 1 mg/kg THC to assess catalepsy, hypothermia and nociception. Doses used were 0.1–10 mg/kg compound or volume-matched vehicle control (1:1:18 ethanol/cremaphor/saline) and assessment of catalepsy (%MPE 60 s) **(A,D)**, body temperature **(B,E)**, and nociception in the tail flick assay (%MPE, 20 s) **(C,F)** were performed; *n* = 5–10/group; data are mean ± SEM. ^*p* < 0.05, ^^^*p* < 0.001 for GAT1665 compared to 1 mg/kg THC; **p < 0.01, ***p < 0.001 for GAT1667 compared to 1 mg/kg THC as determined by two-way ANOVA followed by Bonferroni’s *post-hoc* test.

All enantiomers were evaluated in male C57BL/6 mice using a triad of outcomes consisting of catalepsy, body temperature, and nociception. Previous studies have shown that the racemic GAT591 and GAT593 did not produce catalepsy or hypothermia at 0.1, 1, 3, or 10 mg/kg (i.p.) compared to vehicle (Garai et al., 2020). Both GAT591 and GAT593 however did produce a dose-dependent anti-nociceptive effect in the tail flick assay that was significant relative to vehicle at 3 and 10 mg/kg (Garai et al., 2020). In this study we evaluated all separated optically pure enantiomers in a triad assay. When tested alone, GAT1664, GAT1665, GAT1666 and GAT1667 did not produce catalepsy, hypothermia, or anti-nociceptive effects at 0.1, 1, 3, or 10 mg/kg i.p. compared to vehicle (Figures 5A–C). We also tested these enantiomers as PAMs by co-administering a sub-threshold dose of 1 mg/kg THC with each GAT compound. THC was selected as a common CB1R partial agonist that could produce a moderate *in vivo* response to be modulated by our compounds of interest. It was found that GAT1665 and GAT1667 displayed significant cataleptic, hypothermic, and anti-nociceptive effects at 3 and 10 mg/kg i.p. compared to vehicle (Figures 5D–F). Therefore, GAT1664, GAT1665, GAT1666, and GAT1667 did not affect animal responses to catalepsy, body temperature, or nociception in the warm water tail flick assay in normal, otherwise healthy adult mice as agonists; but GAT1665 and GAT1667 were able to augment THC’s effects *in vivo* when administered once. For comparison, the racemic parent compounds, GAT591 and GAT593, do evoke anti-nociceptive responses *in vivo* when administered alone (Laprairie et al., 2017; Garai et al., 2020).

### 3.4 *In silico* studies

**FIGURE 6:**
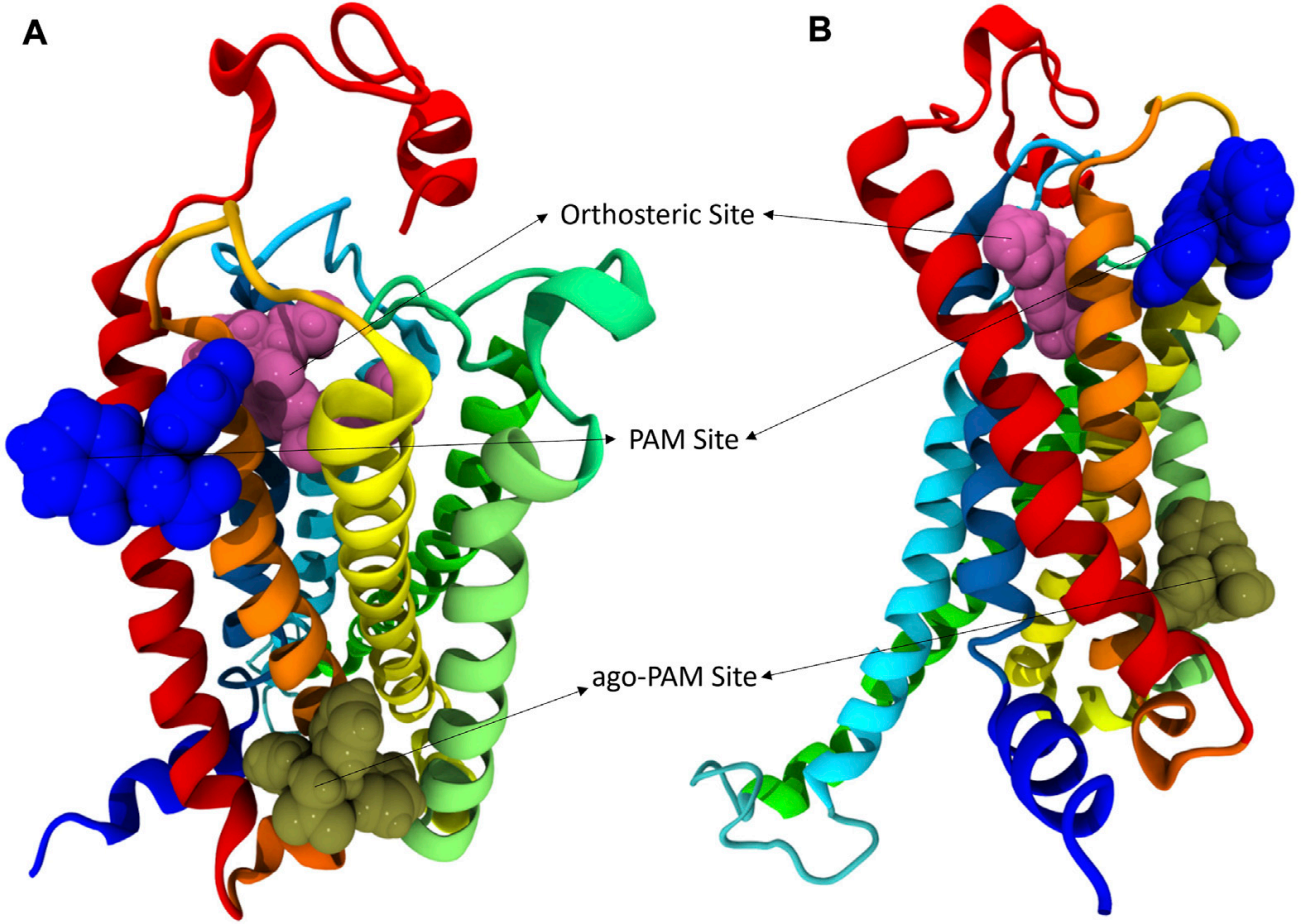
hCB1R with three different binding sites shown. In these views of hCB1R the orthosteric site is shown in magenta, an identified PAM site in blue, and another identified putative ago-PAM site in tan. Transmembrane helices are: I red, II orange, III yellow, IV light green, V green, VI cyan, and VII blue. **(A)** is a visualization of CB1R looking down on the extracellular surface. **(B)** is a visualization of CB1R looking across at the transmembrane domains.

**FIGURE 7:**
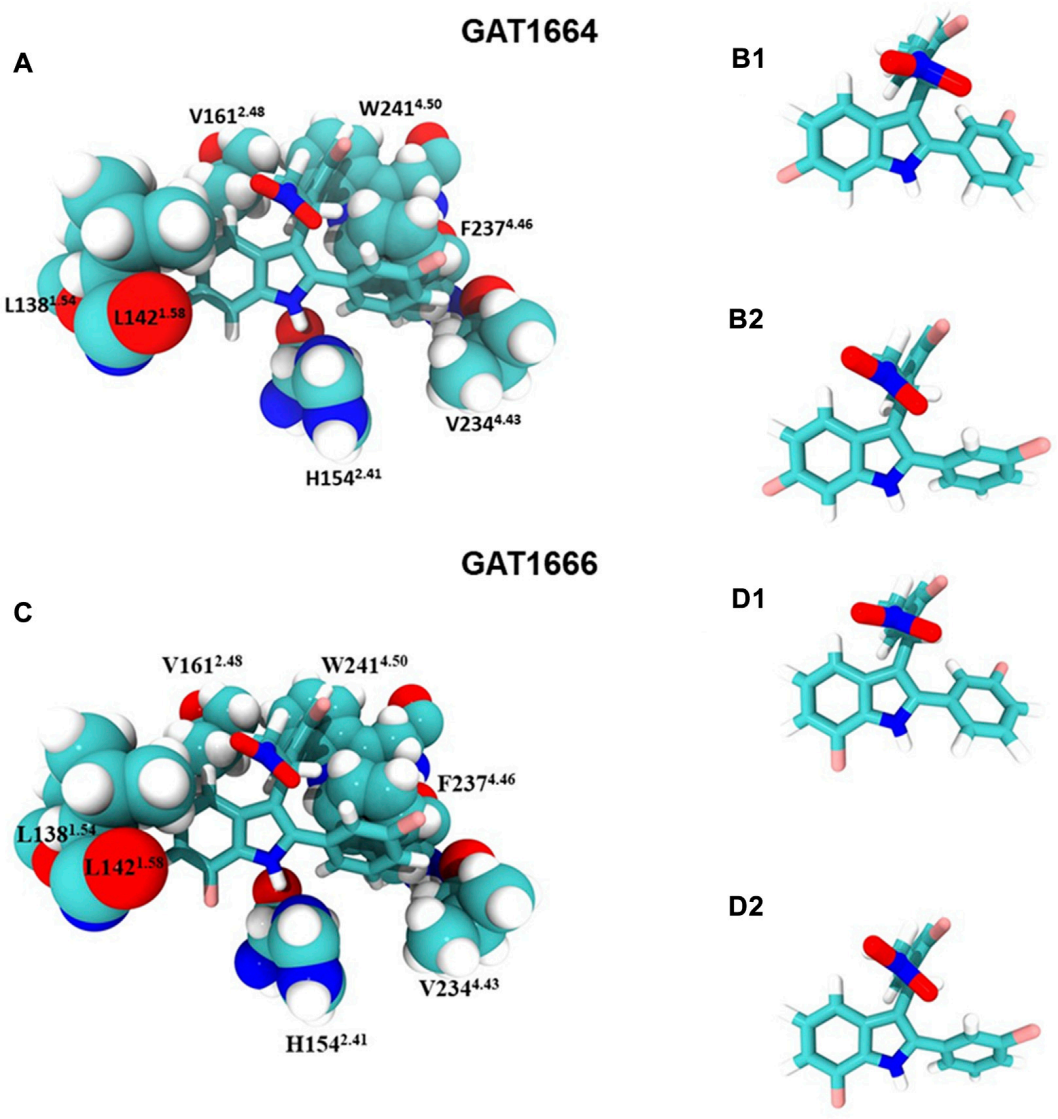
GAT1664 and GAT1666 bind to a putative allosteric agonist site. **(A)** GAT1664 main interactions and the amino acid residues of the binding pocket displayed in tan in Figure 6. The conformation of GAT1664 before it binds to hCB1R **(B1)** and then the conformation of GAT1664.after it binds to hCB1R **(B2)**. **(C)** GAT1666 main interactions and the amino acid residues of the binding pocket displayed in tan in Figure 6. The conformation of GAT1666 before it binds to hCB1R **(D1)** and then the conformation of GAT1664 after it binds to hCB1R **(D2)**.

Compounds were docked to a modelled structure of hCB1R to determine their putative binding sites within the receptor. GAT1664 and GAT1666 displayed the greatest modelled affinity to an allosteric site on hCB1R proximal to the intracellular face of the receptor in tan (Figures 6A,B). An H-bond exists between the indole hydrogen in white and the nitrogen atom in blue (H–N distance 1.96 Å) on H154^2.41^ (Figure 6A). One of the fluorobenzyl groups on GAT has a face-to-face π-π interaction with F237^4.46^ (4.03Å) while the other fluorobenzyl has a T-shaped π-π interaction with each ring on W241^4.50^ (4.96 Å to the 6—membered ring; 5.09 Å to the 5—membered ring) (Figure 6A). Upon binding to hCB1R the conformation of the *ortho-*fluorobenzyl substituent on GAT1664 rotates 90°. The conformational cost for this rotation is 2.35 kcal/mol (Table 1). GAT1666 has the same binding pose, thus the binding energy was measured to look at the relative affinity. GAT1666 is more stable than GAT1664 by 2.31 kcal/mol (∆G GAT1666—∆G GAT1664) which suggests that it has a higher affinity to the allosteric site of hCB1R shown in tan (Figure 6). This can be attributed to changing the fluorine on the sixth position of the indole (GAT1664) to the seventh position on the indole (GAT1666). The binding of a compound to this site may allow it to act as an agonist by promoting the X1 = *g* + → trans conformation of residue F4.46, facilitating breaking of the R3.50/D6.30 ionic lock which has been shown to activate CB1R (Figures 6, 7) (Hurst et al., 2019).

**TABLE 1:** The conformational cost of GAT compounds is displayed as well as the relative affinity represented by binding energy.

Compound	Conformational cost (kcal/mol)	∆G (kcal/mol)
GAT1664	2.35	−69.68
GAT1665	3.27	−60.77
GAT1666	2.48	−71.99
GAT1667	3.60	−64.55

**FIGURE 8:**
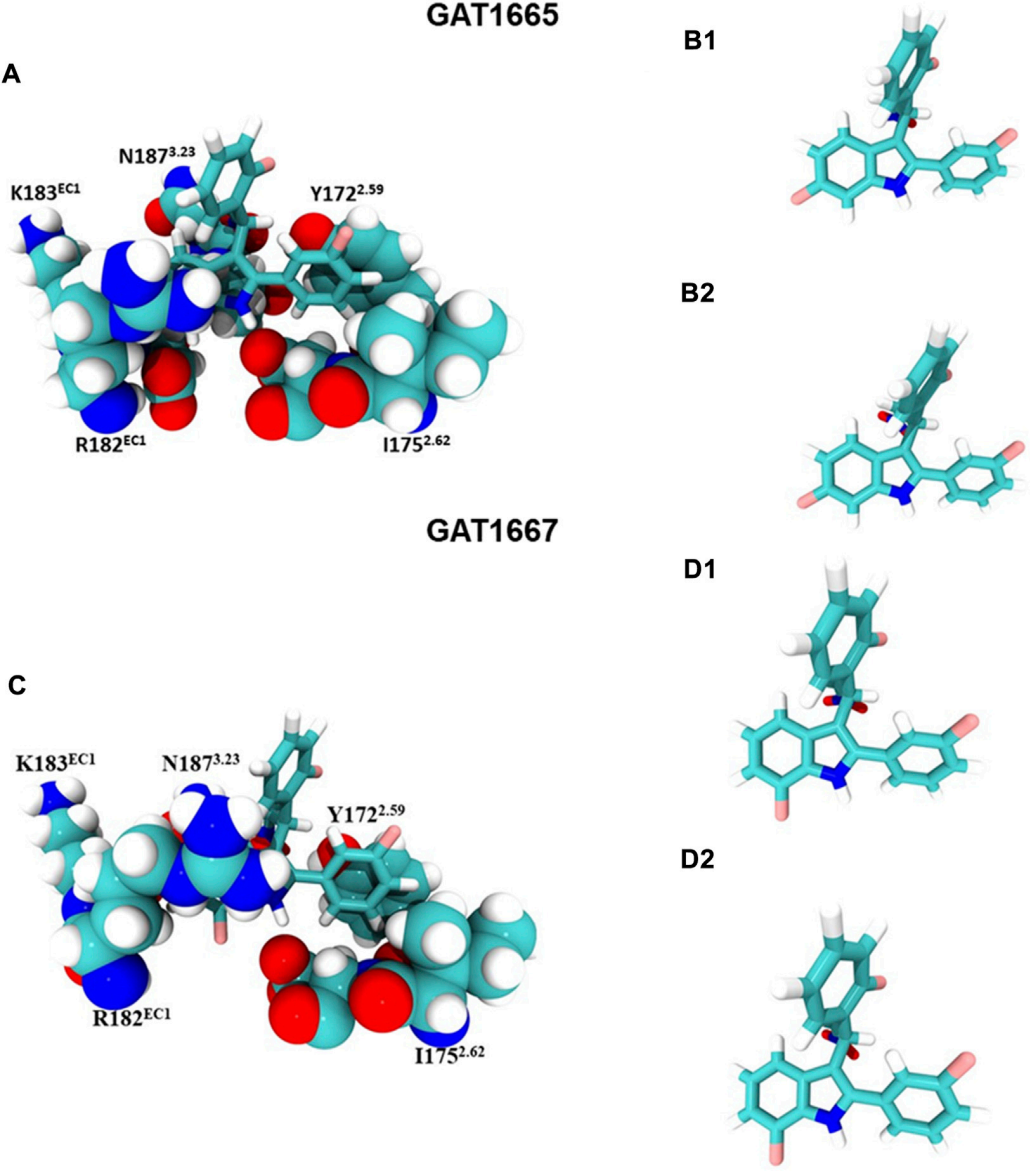
GAT1665 and GAT1667 bind to a putative PAM site. (**A**) GAT1665 main interactions and the amino acid residues of the binding pocket displayed in pink in Figure 6. The conformation of GAT1665 before it binds to hCB1R **(B1)** and then the conformation of GAT1665 after it binds to hCB1R **(B2)**. **(C)** GAT1667 main interactions and the amino acid residues of the binding pocket displayed in pink in Figure 6. The conformation of GAT1667 before it binds to hCB1R **(D1)** and then the conformation of GAT1667 after it binds to hCB1R **(D2)**.

GAT1665 and GAT1667 displayed the greatest modelled affinity to a distinct allosteric site on hCB1R relative to GAT1664 and GAT1666 (Figures 6, 8). The NO_2_ group on GAT1665 has a H-bond between 1 of the oxygens shown in red and a hydrogen atom on Y172^2.59^ (O–H distance 2.17 Å) (Figure 8). Another H-bond exists between the indole hydrogen in white and an oxygen atom in red on D176^2.63^ (H–O distance 2.29 Å) (Figure 8). A cation-π interaction exists between R182^EC1^ and each ring on the indole on GAT1665 (six–membered ring centroid on indole to NH_2_^+^ on R182^EC1^ distance 3.59 Å; five–membered ring centroid on indole to NH_2_^+^ on R182^EC1^ distance 3.66 Å) (Figure 8). One of the fluorobenzyl groups on GAT has a face-to-face π-π interaction with Y172^2.59^ (4.05 Å) (Figure 8). Upon binding to hCB1R the conformation of the NO_2_ rotates in order to H-bond with Y172^2.59^ (Table 1). The conformational cost for this rotation is 3.27 kcal/mol. If we compare the binding energy of GAT1665 and GAT1667 it is apparent that GAT1667 is more stable than GAT1665 bound to the PAM site by 4.78 kcal/mol (∆G GAT1667—∆G GAT1665). This can be attributed to changing the fluorine on the sixth position of the indole (GAT1665) to the seventh position on the indole (GAT1667). The previously characterized compound GAT229 also binds to this site and lacks intrinsic activity thus both these compounds alone cannot induce the active state of hCB1R and thus are PAMs. The *R*-enantiomers (GAT1665 and GAT1667) undergo a smaller conformational cost than the *S*-enantiomers (GAT1664 and GAT1666) upon binding to their respective sites on hCB1R (Table 1). The *R*-enantiomers (GAT1665 and GAT1667) bind to the ago-PAM site (∆G = −60.77 and −64.55 kcal/mol) significantly weaker than the *S*-enantiomers (GAT1664 and GAT1666) bind to the PAM site (−69.68 and −71.99 kcal/mol). Of note, binding of enantiomers to either site was not absolute but was more energetically favourable for the *S-*enantiomers to bind near the intracellular surface and the *R*-enantiomers to bind near the first extracellular loop.

## 4 Conclusion

In summary, the purified enantiomers of GAT591 and GAT593 displayed unique pharmacology. The purified enantiomers GAT1664, GAT1665, GAT1666, and GAT1667 bound to hCB1R in the low nM range and were shown to enhance binding of [^3^H]CP55,940 at hCB1R. Enhancing the binding of an orthosteric ligand is a characteristic of a PAM and these observations are consistent with all compounds operating in part *via* positive allostery and/or non-competitive allosteric agonism (Alaverdashvili and Laprairie, 2018; Kenakin and Strachan, 2018). Future explorations of these compounds will utilize full concentration-response curves of these compounds with and without CP55,940 or an endogenous orthosteric agonist (e.g. 2-AG). Such more in-depth experiments will allow for the use an operational model of allosterism that quantifies co-operativity effects on potency, efficacy, and bias as our group has done previously for both PAMs and NAMs (Laprairie et al., 2016; Laprairie et al., 2017); and determine whether the tested compounds display probe-dependence.

In addition to this, we demonstrated that these enantiomers activate hCB1R-dependent inhibition of cAMP and βarrestin2 recruitment. All of the GAT compounds tested here inhibited cAMP accumulation as allosteric agonists and PAMs (i.e. ago-PAMs), with GAT1665 and GAT1667 displaying the greatest potency and efficacy as CB1R PAMs in the presence of 100 nM CP55,940. In the βarrestin2 assay, GAT1665 and GAT1667 (*R*-enantiomers) acted as ago-PAM’s while GAT1664 and GAT1666 (*S*-enantiomers) showed little to no activity as either an agonist or PAM. In the G protein coupling assay, the *R*-enantiomers GAT1665 and GAT1667 also showed higher potency and efficacy than *S*-enantiomers as agonists and PAMs. As for the *in vivo* studies, no compounds tested produced catalepsy, hypothermia at or antinociceptive effects at 0.1, 1, 3 or 10 mg/kg i.p. compared to vehicle when tested alone, but GAT1665 and GAT1667 did potentiate the effects of THC as ago-PAMs. These data are congruent with previous observations that CB1R ago-PAMs are inactive or minimally active *in vivo* in wild-type, otherwise healthy animals under non-pathological and acute conditions (Alaverdashvili and Laprairie, 2018). In planned future studies, these ago-PAMs will be further assessed in rodent models of absence epilepsy and pain to determine whether they are able to augment the endogenous cannabinoid signaling as we observed previously with related allosteric ligands (Slivicki et al., 2020; Roebuck et al., 2021). In addition, the magnitude of observed *in vivo* effects may have been effected by pharmacokinetics of our compounds; planned future studies will assess and optimize the pharmacokinetics of CB1R allosteric ligands. Our *in silico* data support *in vitro* binding and signaling data that indicate these compounds interact with an allosteric site on CB1R that is distinct from the primary orthosteric agonist binding site (Price et al., 2005; Vigolo et al., 2015; Hua et al., 2017; Krishna Kumar et al., 2019). GAT1664 and GAT1666 preferentially bind to a putative allosteric agonist site of hCB1R near the intracellular face of the receptor between transmembrane helices I and IV; while GAT1665 and GAT1667 preferentially bind to a putative PAM site of CB1R on the extracellular receptor surface. Enantiomers may display low affinity to multiple allosteric sites, accounting for the allosteric agonist activity of GAT1665 and GAT1667 (Price et al., 2005; Kapur et al., 2007; Hurst et al., 2019). However, future dynamic modeling and crystallization studies will be required to fully understand how the putative allosteric sites contribute to partial agonism and positive allosteric modulation, as the current models represent static interactions. Collectively, these data support retained, potent, G protein-selective, ago-PAM activity at hCB1R for the GAT211 ligand scaffold and demonstrate enhanced potency and efficacy of these ligands relative to the parent compound (Laprairie et al., 2017; Garai et al., 2020). Moreover, recent data from the racemic mixtures of these enantiomers indicates fluorine addition improves both metabolic stability and blood brain barrier penetrance; with GAT593—and therefore GAT1667—being superior to GAT591 (Garai et al., 2020). Therefore, studies are now underway to assess the *in vivo* efficacy of GAT1667 in the contexts of absence epilepsy and pain during both acute and chronic treatment paradigms.

### 4.1 Permission to reuse and copyright

This is an open-access article distributed under the terms of the Creative Commons Attribution License (CC BY). The use, distribution or reproduction in other forums is permitted, provided the original author(s) and the copyright owner(s) are credited and that the original publication in this journal is cited, in accordance with accepted academic practice. No use, distribution or reproduction is permitted which does not comply with these terms.

## Data Availability

The original contributions presented in the study are included in the article/Supplementary Material, further inquiries can be directed to the corresponding authors.

## References

[B1] Alaverdashvili M. Laprairie R. B. (2018). The future of type 1 cannabinoid receptor allosteric ligands. Drug Metab. Rev. 50, 14–25. 10.1080/03602532.2018.1428341 29355038

[B2] Black J. W. Leff P. Shankley N. P. Wood J. (2010). An operational model of pharmacological agonism : The effect of E/[ A ] curve shape on agonist dissociation constant estimation. Br. J. Pharmacol. 160, S54–S64. 10.1111/j.1476-5381.2010.00855.x 20590656 PMC2947418

[B3] Carrera J. Tomberlin J. Kurtz J. Karakaya E. Bostanciklioglu M. Albayram O. (2020). Endocannabinoid signaling for GABAergic-microglia (mis)communication in the brain aging. Front. Neurosci. 14, 606808. 10.3389/fnins.2020.606808 33613174 PMC7887316

[B4] Clark M. Guarnieri F. Shkurko I. Wiseman J. (2006). Grand canonical Monte Carlo simulation of ligand-protein binding. J. Chem. Inf. Model. 46, 231–242. 10.1021/ci050268f 16426059

[B5] Di Marzo V. (2018). New approaches and challenges to targeting the endocannabinoid system. Nat. Rev. Drug Discov. 17, 623–639. 10.1038/nrd.2018.115 30116049

[B6] Dopart R. Lu D. Lichtman A. H. Kendall D. A. (2018). Allosteric modulators of cannabinoid receptor 1: Developing compounds for improved specificity. Drug Metab. Rev. 50, 3–13. 10.1080/03602532.2018.1428342 29355030 PMC6134837

[B7] Estrada J. A. Contereras I. (2020). Endocannabinoid receptors in the CNS: Potential drug targets for the prevention and treatment of neurologic and psychiatric disorders. Curr. Neuropharmacol. 18, 769–787. 10.2174/1570159X18666200217140255 32065105 PMC7536826

[B8] Fay J. F. Farrens D. L. (2015). Structural dynamics and energetics underlying allosteric inactivation of the cannabinoid receptor CB1. Proc. Natl. Acad. Sci. U. S. A. 112, 8469–8474. 10.1073/pnas.1500895112 26100912 PMC4500223

[B9] Fay J. F. Farrens D. L. (2013). The membrane proximal region of the cannabinoid receptor CB1 N-terminus can allosterically modulate ligand affinity. Biochemistry 52, 8286–8294. 10.1021/bi400842k 24206272 PMC3938390

[B10] Fiser A. Sali A. (2003). ModLoop: Automated modeling of loops in protein structures. Bioinformatics 19, 2500–2501. 10.1093/bioinformatics/btg362 14668246

[B11] Garai S. Kulkarni P. M. Schaffer P. C. Leo L. M. Brandt A. L. Zagzoog A. (2020). Application of fluorine- and nitrogen-walk approaches: Defining the structural and functional diversity of 2-phenylindole class of cannabinoid 1 receptor positive allosteric modulators. J. Med. Chem. 63, 542–568. 10.1021/acs.jmedchem.9b01142 31756109 PMC7077750

[B12] Garai S. Leo L. M. Szczesniak A. M. Hurst D. P. Schaffer P. C. Zagzoog A. (2021). Discovery of a biased allosteric modulator for cannabinoid 1 receptor: Preclinical anti-glaucoma efficacy. J. Med. Chem. 64, 8104–8126. 10.1021/acs.jmedchem.1c00040 33826336

[B46] Greig I. R. Baillie G. L. Abdelrahman M. Trembleau L. Ross R. A. (2016). Development of indole sulfonamides as cannabinoid receptor negative allosteric modulators. Bioorg. Med. Chem. Lett. 26, 4403–4407. 10.1016/j.bmcl.2016.08.018 27542310

[B13] Guarnieri F. Mezei M. (1996). Simulated annealing of chemical potential: A general procedure for locating bound waters. Application to the study of the differential hydration propensities of the major and minor grooves of DNA. J. Am. Chem. Soc. 118, 8493–8494. 10.1021/ja961482a

[B14] Hryhorowicz S. Kaczmarek-Rys M. Andrzejewska A. Staszak K. Hryhorowicz M. Korcz A. (2019). Allosteric modulation of cannabinoid receptor 1-current challenges and future opportunities. Int. J. Mol. Sci. 20, 5874. 10.3390/ijms20235874 PMC6928801 31771126

[B15] Hua T. Vemuri K. Nikas S. P. Laprairie R. B. Wu Y. Qu L. (2017). Crystal structures of agonist-bound human cannabinoid receptor CB1. Nature 547, 468–471. 10.1038/nature23272 28678776 PMC5793864

[B16] Hurst D. P. Garai S. Kulkarni P. M. Schaffer P. C. Reggio P. H. Thakur G. A. (2019). Identification of Cb1 receptor allosteric sites using force-biased mmc simulated annealing and validation by structure-activity relationship studies. ACS Med. Chem. Lett. 10, 1216–1221. 10.1021/acsmedchemlett.9b00256 31413808 PMC6691559

[B17] Iannotti F. A. Di Marzo V. Petrosino S. (2016). Endocannabinoids and endocannabinoid-related mediators: Targets, metabolism and role in neurological disorders. Prog. Lipid Res. 62, 107–128. 10.1016/j.plipres.2016.02.002 26965148

[B18] Kapur A. Hurst D. P. Fleischer D. Whitnell R. Thakur G. A. Makriyannis A. (2007). Mutation studies of Ser7.39 and Ser2.60 in the human CB1 cannabinoid receptor: Evidence for a serine-induced bend in CB1 transmembrane helix 7. Mol. Pharmacol. 71, 1512–1524. 10.1124/mol.107.034645 17384224

[B19] Kenakin T. Strachan R. T. (2018). PAM-antagonists: A better way to block pathological receptor signaling? Trends Pharmacol. Sci. 39, 748–765. 10.1016/j.tips.2018.05.001 29885909

[B20] Kenakin T. Watson C. Muniz-Medina V. Christopoulos A. Novick S. A. (2012). A simple method for quantifying functional selectivity and agonist bias. ACS Chem. Neurosci. 3, 193–203. 10.1021/cn200111m 22860188 PMC3369801

[B21] Kilkenny C. Browne W. J. Cuthill I. C. Emerson M. Altman D. G. (2010). Improving bioscience research reporting: The arrive guidelines for reporting animal research. PLoS Biol. 8, 10004122–e1000510. 10.1371/journal.pbio.1000412 PMC2893951 20613859

[B22] Krishna Kumar K. Shalev-Benami M. Robertson M. J. Hu H. Banister S. D. Hollingsworth S. A. (2019). Structure of a signaling cannabinoid receptor 1-G protein complex. Cell 176, 448–458. 10.1016/j.cell.2018.11.040 30639101 PMC6461403

[B23] Laprairie R. B. Bagher A. M. Kelly M. E. Denovan-Wright E. M. (2016). Biased type 1 cannabinoid receptor signaling influences neuronal viability in a cell culture model of Huntington disease. Mol. Pharmacol. 89, 364–375. 10.1124/mol.115.101980 26700564

[B24] Laprairie R. B. Bagher A. M. Rourke J. L. Zrein A. Cairns E. A. Kelly M. E. M. (2019). Positive allosteric modulation of the type 1 cannabinoid receptor reduces the signs and symptoms of Huntington’s disease in the R6/2 mouse model. Neuropharmacology 151, 1–12. 10.1016/j.neuropharm.2019.03.033 30940536 PMC6544167

[B25] Laprairie R. B. Kulkarni P. M. Deschamps J. R. Kelly M. E. M. Janero D. R. Cascio M. G. (2017). Enantiospecific allosteric modulation of cannabinoid 1 receptor. ACS Chem. Neurosci. 8, 1188–1203. 10.1021/acschemneuro.6b00310 28103441 PMC13094344

[B26] Leo L. M. Abood M. E. (2021). CB1 cannabinoid receptor signaling and biased signaling. Molecules 26, 5413. 10.3390/molecules26175413 34500853 PMC8433814

[B27] Lutz B. (2020). Neurobiology of cannabinoid receptor signaling. Dialogues Clin. Neurosci. 22, 207–222. 10.31887/DCNS.2020.22.3/blutz 33162764 PMC7605026

[B28] Marcu J. Shore D. M. Kapur A. Trznadel M. Makriyannis A. Reggio P. H. (2013). Novel insights into CB1 cannabinoid receptor signaling: A key interaction identified between the extracellular-3 loop and transmembrane helix 2. J. Pharmacol. Exp. Ther. 345, 189–197. 10.1124/jpet.112.201046 23426954 PMC3629795

[B29] Mezei M. (2011). Mmc: A Monte Carlo and analysis program. Biophys. J. 100, 157a. 10.1016/j.bpj.2010.12.1075

[B30] Mielnik C. A. Lam V. M. Ross R. A. (2021). CB1 allosteric modulators and their therapeutic potential in CNS disorders. Prog. Neuropsychopharmacol. Biol. Psychiatry 106, 110163. 10.1016/j.pnpbp.2020.110163 33152384

[B31] Mitjavila J. Yin D. Kulkarni P. M. Zanato C. Thakur G. A. Ross R. (2018). Enantiomer-specific positive allosteric modulation of CB1 signaling in autaptic hippocampal neurons. Pharmacol. Res. 129, 475–481. 10.1016/j.phrs.2017.11.019 29158048 PMC5828971

[B32] Morales P. Goya P. Jagerovic N. Hernandez-Folgado L. (2016). Allosteric modulators of the CB1 cannabinoid receptor: A structural update review. Cannabis Cannabinoid Res. 1, 22–30. 10.1089/can.2015.0005 28861476 PMC5576597

[B33] Patel M. Finlay D. B. Glass M. (2021). Biased agonism at the cannabinoid receptors - evidence from synthetic cannabinoid receptor agonists. Cell. Signal. 78, 109865. 10.1016/j.cellsig.2020.109865 33259937

[B34] Perez-Olives C. Rivas-Santisteban R. Lillo J. Navarro G. Franco R. (2021). Recent advances in the potential of cannabinoids for neuroprotection in Alzheimer’s, Parkinson’s, and Huntington’s diseases. Adv. Exp. Med. Biol. 1264, 81–92. 10.1007/978-3-030-57369-0_6 33332005

[B35] Price M. R. Baillie G. L. Thomas A. Stevenson L. A. Easson M. Goodwin R. (2005). Allosteric modulation of the cannabinoid CB1 receptor. Mol. Pharmacol. 68, 1484–1495. 10.1124/mol.105.016162 16113085

[B36] Roebuck A. J. Greba Q. Onofrychuk T. J. McElroy D. L. Sandini T. M. Zagzoog A. (2020). Dissociable changes in spike and wave discharges following exposure to injected cannabinoids and smoked Cannabis in genetic absence epilepsy rats from strasbourg. Eur. J. Neurosci. 55, 1063–1078. 10.1111/ejn.15096 33370468

[B37] Roebuck A. J. Greba Q. Smolyakova A. M. Alaverdashvili M. Marks W. N. Garai S. (2021). Positive allosteric modulation of type 1 cannabinoid receptors reduces spike-and-wave discharges in genetic absence epilepsy rats from strasbourg. Neuropharmacology 190, 108553. 10.1016/j.neuropharm.2021.108553 33845076

[B38] Shim J. Y. Bertalovitz A. C. Kendall D. A. (2011). Identification of essential cannabinoid-binding domains: Structural insights into early dynamic events in receptor activation. J. Biol. Chem. 286, 33422–33435. 10.1074/jbc.M111.261651 21795705 PMC3190901

[B39] Slivicki R. A. Iyer V. Mali S. S. Garai S. Thakur G. A. Crystal J. D. (2020). Positive allosteric modulation of CB(1) cannabinoid receptor signaling enhances morphine antinociception and attenuates morphine tolerance without enhancing morphine-induced dependence or reward. Front. Mol. Neurosci. 13, 54. 10.3389/fnmol.2020.00054 32410959 PMC7199816

[B40] Song Z. H. Bonner T. I. (1996). A lysine residue of the cannabinoid receptor is critical for receptor recognition by several agonists but not WIN55212-2. Mol. Pharmacol. 49, 891–896. 8622639

[B41] Spartan (2018). Spartan’18 parallel suite. Irvine, CA: Wavefunction.

[B42] Vigolo A. Ossato A. Trapella C. Vincenzi F. Rimondo C. Seri C. (2015). Novel halogenated derivates of JWH-018: Behavioral and binding studies in mice. Neuropharmacology 95, 68–82. 10.1016/j.neuropharm.2015.02.008 25769232

[B43] Wang X. Li Y. Gao Y. Yang Z. Lu C. Zhu T. (2018). A quantum mechanical computational method for modeling electrostatic and solvation effects of protein. Sci. Rep. 8, 5475–5510. 10.1038/s41598-018-23783-8 29615707 PMC5882933

[B44] Zagzoog A. Brandt A. L. Black T. Kim E. D. Burkart R. Patel M. (2021). Assessment of select synthetic cannabinoid receptor agonist bias and selectivity between the type 1 and type 2 cannabinoid receptor. Sci. Rep. 11, 10611. 10.1038/s41598-021-90167-w 34012003 PMC8134483

[B45] Zhang J. Chen Q. Liu B. (2020). iDRBP_MMC: Identifying DNA-binding proteins and RNA-binding proteins based on multi-label learning model and motif-based convolutional neural network. J. Mol. Biol. 432, 5860–5875. 10.1016/j.jmb.2020.09.008 32920048

