# Supplementary material for: Pharmacological evaluation of enantiomerically separated positive allosteric modulators of cannabinoid 1 receptor, GAT591 and GAT593
Source: Front Pharmacol. 2022 Oct 25;13:919605. doi: 10.3389/fphar.2022.919605 (PMC9640980; doi:10.3389/fphar.2022.919605)
Supplement: Supplementary file 1 [file Table1.DOCX]

**Supporting Information for:**

**Pharmacological Evaluation of Enantiomerically Separated Positive Allosteric Modulators of Cannabinoid 1 Receptor, GAT591 and GAT593**

Asher L. Brandt^1^, Sumanta Garai^2^, Ayat Zagzoog^1^, Dow P. Hurst^3^, Lesley A. Stevenson^4^, Roger G. Pertwee^4^, Gregory H. Imler^5^, Patricia H. Reggio^3^, Ganesh A. Thakur^2^* and Robert B. Laprairie^1,6^*

**Synthesis, Chiral Separation and Absolute stereochemistry Determination:** We synthesized **GAT591** and **GAT593** on a multigram scale using our previously published method (Ref: *J. Med. Chem.* 2020, 63, 542–568). Both enantiomers of each compound were separated in high optical purity (>99%) using superfluid chiral HPLC (for details see supporting information). The absolute stereochemistry of **(+)-GAT1664** and **(+)-GAT1666** was determined by single-crystal X-ray diffraction technique (for details see supporting formation) and was found to be “S” for both of these enantiomers (Figure 1). Based on this study, we can predict the absolute stereochemistry of each opposite enantiomers, **(-)-GAT1665** and **(-)-GAT1667** as “R” (Figures 1 and 2).


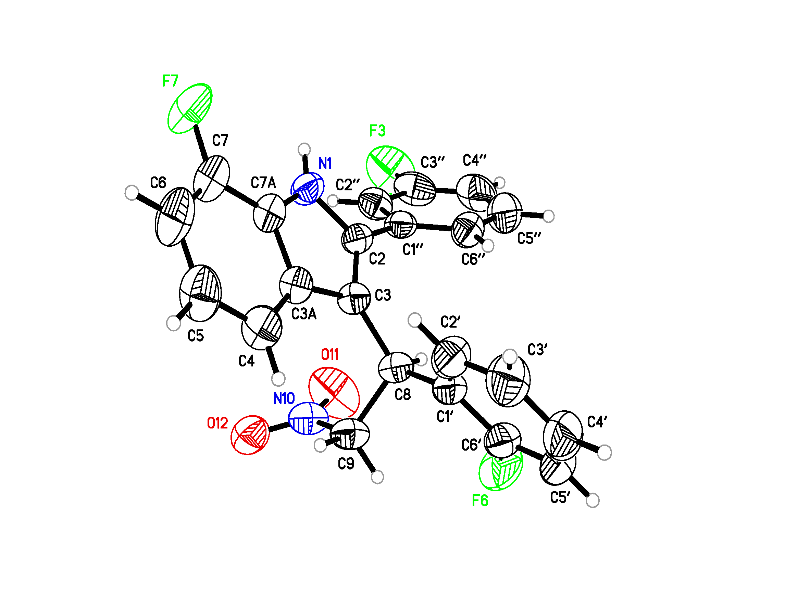


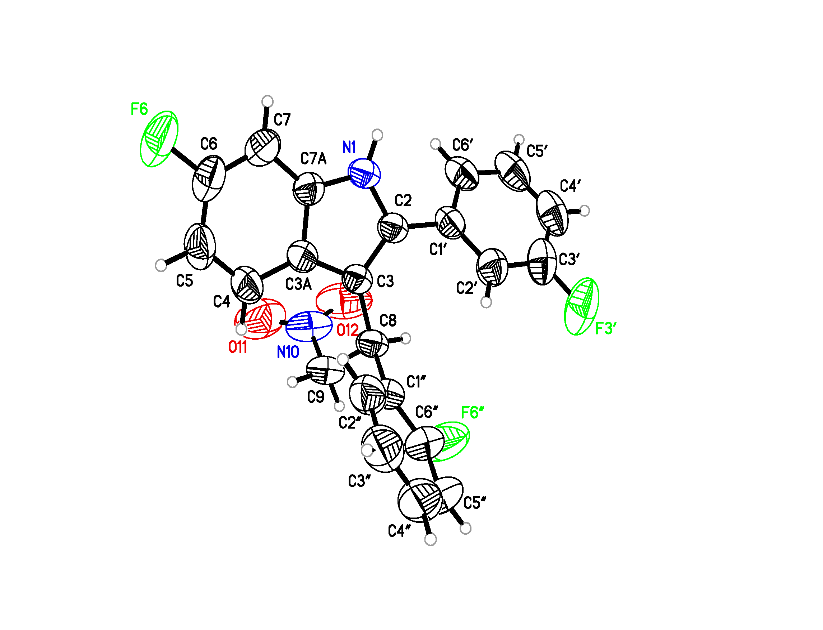


**a)**

**b)**

Supplementary Figure 1. ORTEP diagram of **(+)-GAT1664** (**a**, CCDC no. 2086717) and **(+)-GAT1666** (**b**, CCDC no. 2086718).

**Supporting Information:**

**(+)-(*S)*-6-Fluoro-2-(3-fluorophenyl)-3-(1-(2-fluorophenyl)-2-nitroethyl)-1*H*-indole (GAT1664)**: ^1^H NMR (400 MHz, DMSO-d_6_): δ 11.66 (bs, 1H), 7.77 (dd, *J*= 5.3, 8.8 Hz, 1H), 7.67−7.57 (m, 2H), 7.39−7.25 (m, 4H), 7.18−7.89 (m, 3H), 6.85 (t, *J*= 8.7 Hz, 1H,), 5.59-5.53 (m, 2H), 5.43 (d, *J*= 8.4 Hz, 1H).; [α]_D_ ^24^ = +70.80 (c 1 g/100 mL, MeOH); MS-ESI (*m/z*) 397 [M + H] ^+^.

**(-)-(*R)*-6-Fluoro-2-(3-fluorophenyl)-3-(1-(2-fluorophenyl)-2-nitroethyl)-1*H*-indole (GAT1665)**: ^1^H NMR (400 MHz, DMSO-d_6_): δ 11.66 (bs, 1H), 7.77 (dd, *J*= 5.3, 8.8 Hz, 1H), 7.67−7.57 (m, 2H), 7.39−7.25 (m, 4H), 7.18−7.89 (m, 3H), 6.85 (t, *J*= 8.7 Hz, 1H,), 5.59-5.53 (m, 2H), 5.43 (d, *J*= 8.4 Hz, 1H).; [α]_D_ ^24^ = -87.85 (c 1 g/100 mL, MeOH) ; MS-ESI (*m/z*) 397 [M + H] ^+^.

**(+)-(*S)*-7-Fluoro-2-(3-fluorophenyl)-3-(1-(2-fluorophenyl)-2-nitroethyl)-1H-indole (GAT1666)**: ^1^H NMR (400 MHz, DMSO-d_6_): δ 11.96 (bs, 1H), 7.67-7.57 (m, 3H), 7.41−7.25 (m, 4H), 7.18−6.98 (m, 2H), 6.96 (d, *J*= 8.0 Hz, 1H,), 5.61-5.49 (m, 2H), 5.393 (t, *J*= 8.4 Hz, 1H).; [α]_D_ ^24^ = +24.80 (c 1 g/100 mL, MeOH); MS-ESI (*m/z*) 397 [M + H] ^+^.

**(-)-(*R)*-7-Fluoro-2-(3-fluorophenyl)-3-(1-(2-fluorophenyl)-2-nitroethyl)-1H-indole (GAT1667)**: ^1^H NMR (400 MHz, DMSO-d_6_): δ 11.96 (bs, 1H), 7.67-7.57 (m, 3H), 7.41−7.25 (m, 4H), 7.18−6.98 (m, 2H), 6.96 (d, *J*= 8.0 Hz, 1H,), 5.61-5.49 (m, 2H), 5.393 (t, *J*= 8.4 Hz, 1H).; [α]_D_ ^24^ = -75.86 (c 1 g/100 mL, MeOH); MS-ESI (*m/z*) 397 [M + H] ^+^.

Preparative HPLC chromatogram for (+)-GAT1664


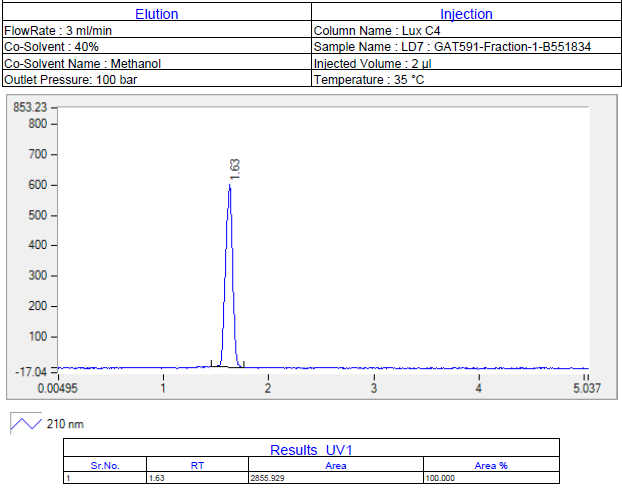


Preparative HPLC chromatogram for (-)-GAT1665


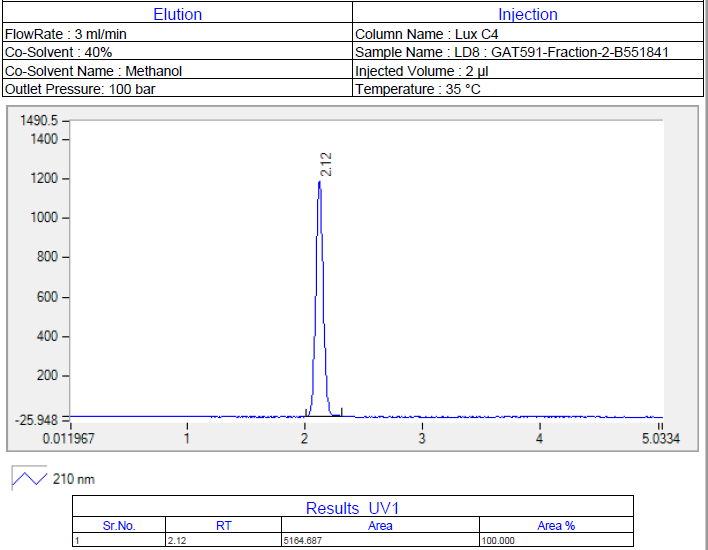


Preparative HPLC chromatogram for (+)-GAT1666


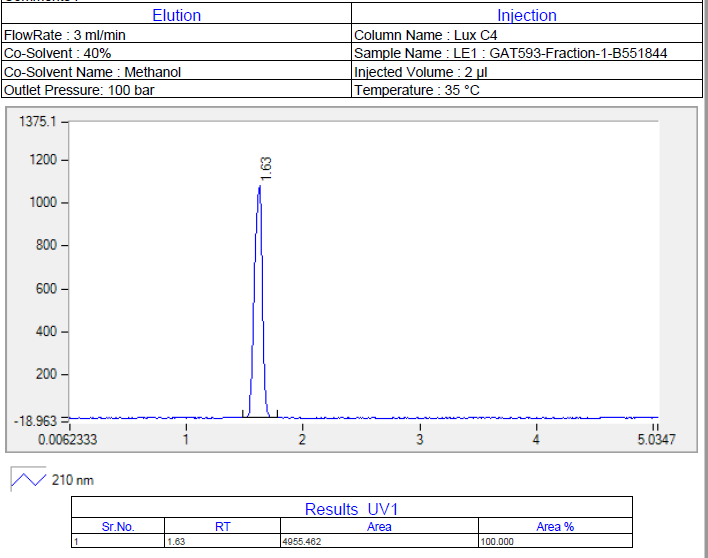


Preparative HPLC chromatogram for (-)-GAT1667


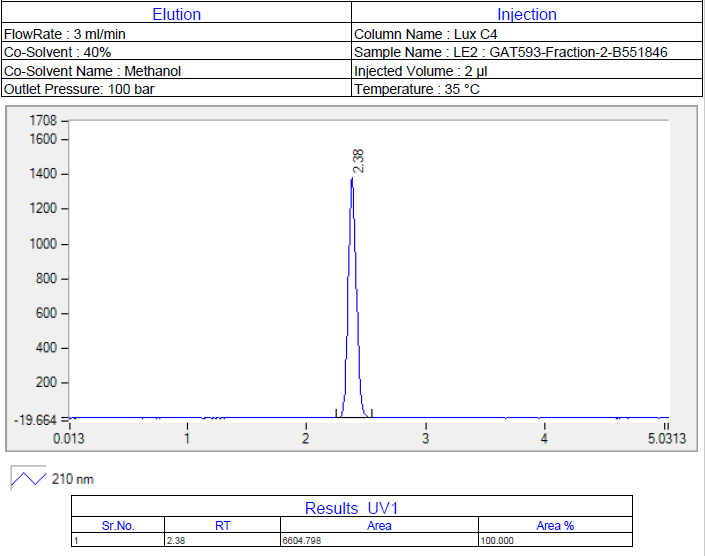


**Single-crystal X-ray Diffraction Analysis of GAT1664.**

C_22_H_15_F_3_N_2_O_2_, FW = 396.36, orthorhombic, P2_1_2_1_2_1_, a = 7.188(2) Å, b = 12.229(3) Å, c = 21.562(6) Å, *α* = 90°, *β* = 90°, *γ* = 90°, V = 1895.4(9) Å^3^, Z = 4, *ρ*calc (293K) = 1.389 Mg/m^3^, *μ* = 0.936 mm^-1^, *F*(000) = 816, R_1_ = 0.0352 for 3524 observed (I > 2σI) reflections and 0.0389 for all 3832 reflections, Goodness-of-fit = 1.042, 293 parameters.

A clear colorless plate crystal of dimensions 0.356 x 0.070 x 0.026 mm was mounted on a MiteGen MicroMesh using a small amount of Cargille Immersion Oil. Data were collected on a Bruker three-circle platform diffractometer equipped with a PHOTON II CPAD detector. The crystals were irradiated using a 1μs microfocus CuK_α_ source (λ = 1.54178) with Montel optics. Data was collected at room temperature (20°C).

Data collection was performed and the unit cell was initially refined using *APEX3* [v2015.5-2].^1^ Data Reduction was performed using *SAINT* [v8.34A]^2^ and *XPREP* [v2014/2]^3^. Corrections were applied for Lorentz, polarization, and absorption effects using *SADABS* [v2014/2].^4^ The structure was solved and refined with the aid of the program SHELXL-2014/7.^5^ The full-matrix least-squares refinement on F^2^ included atomic coordinates and anisotropic thermal parameters for all non-H atoms. Hydrogen atoms were located from the difference electron-density maps and added using a riding model.

**Table 1**. Crystal data and structure refinement for GAT1664.

Identification code GAT1664

Empirical formula C_22_H_15_F_3_N_2_O_2_

Formula weight 396.36

Temperature 293(2) K

Wavelength 1.54178 Å

Crystal system Orthorhombic

Space group P2_1_2_1_2_1_

Unit cell dimensions a = 7.188(2) Å α= 90°.

b = 12.229(3) Å β= 90°.

c = 21.562(6) Å γ = 90°.

Volume 1895.4(9) Å3

Z 4

Density (20°C) 1.389 Mg/m3

Absorption coefficient 0.936 mm-1

F(000) 816

Crystal size 0.356 x 0.070 x 0.026 mm3

Theta range for data collection 4.156 to 74.477°.

Index ranges -6<=h<=8, -15<=k<=15, -26<=l<=26

Reflections collected 15482

Independent reflections 3832 [R_int_ = 0.0252]

Completeness to theta = 67.679° 99.6 %

Absorption correction Semi-empirical from equivalents

Max. and min. transmission 1.0000 and 0.8485

Refinement method Full-matrix least-squares on F2

Data / restraints / parameters 3832 / 86 / 293

Goodness-of-fit on F2 1.042

Final R indices [I>2sigma(I)] R_1_ = 0.0352, wR_2_ = 0.0963

R indices (all data) R1 = 0.0389, wR_2_ = 0.1000

Absolute structure parameter 0.00(5)

Largest diff. peak and hole 0.109 and -0.140 e.Å-3

**Table 2**. Atomic coordinates (x 104) and equivalent isotropic displacement parameters (Å2x 103)

for GAT1664. U(eq) is defined as one third of the trace of the orthogonalized Uij tensor.

________________________________________________________________________________

x y z U(eq)

________________________________________________________________________________

N(1) 6926(3) 7345(1) 5407(1) 56(1)

C(2) 6582(3) 6284(2) 5221(1) 50(1)

C(3) 5941(3) 5694(2) 5718(1) 51(1)

C(3A) 5907(3) 6427(2) 6240(1) 52(1)

C(4) 5427(4) 6338(2) 6872(1) 65(1)

C(5) 5615(4) 7230(2) 7254(1) 74(1)

C(6) 6282(4) 8202(2) 7014(1) 72(1)

F(6) 6472(3) 9065(2) 7410(1) 105(1)

C(7) 6753(4) 8357(2) 6409(1) 64(1)

C(7A) 6543(3) 7447(2) 6025(1) 53(1)

C(8) 5274(3) 4525(2) 5675(1) 57(1)

C(9) 3344(4) 4354(2) 5971(1) 68(1)

N(10) 1980(4) 5167(2) 5735(2) 87(1)

O(11) 1011(4) 5646(2) 6121(2) 132(1)

O(12) 1922(4) 5341(2) 5182(1) 123(1)

C(1') 6867(3) 5978(2) 4570(1) 53(1)

C(2') 7574(4) 4960(2) 4409(1) 66(1)

C(3') 7746(6) 4688(4) 3797(2) 78(3)

F(3') 8373(4) 3682(2) 3646(1) 119(1)

C(3B) 7789(12) 4685(15) 3772(6) 78(14)

C(4') 7294(4) 5404(3) 3323(1) 81(1)

C(5') 6652(6) 6401(4) 3488(2) 78(3)

C(5B) 6597(17) 6440(12) 3470(7) 83(18)

F(3B) 6020(20) 7128(13) 3048(5) 122(5)

C(6') 6411(3) 6710(2) 4098(1) 64(1)

C(1") 6581(4) 3680(2) 5952(1) 63(1)

C(2") 7827(4) 3906(2) 6423(1) 74(1)

C(3") 8950(5) 3091(3) 6678(2) 96(1)

C(4") 8832(6) 2043(3) 6454(2) 117(2)

C(5") 7655(7) 1795(3) 5974(2) 118(1)

C(6") 6565(5) 2620(2) 5734(2) 89(1)

F(6") 5399(5) 2369(1) 5258(1) 127(1)

________________________________________________________________________________**Table 3**. Bond lengths [Å] and angles [°] for GAT1664.

_____________________________________________________

N(1)-C(7A) 1.366(3) N(1)-C(2) 1.380(3)

N(1)-H(1) 0.86(3) C(2)-C(3) 1.371(2)

C(2)-C(1') 1.467(3) C(3)-C(3A) 1.440(3)

C(3)-C(8) 1.511(3) C(3A)-C(7A) 1.407(3)

C(3A)-C(4) 1.408(3) C(4)-C(5) 1.373(3)

C(4)-H(4) 0.9300 C(5)-C(6) 1.382(4)

C(5)-H(5) 0.9300 C(6)-C(7) 1.361(4)

C(6)-F(6) 1.364(3) C(7)-C(7A) 1.395(3)

C(7)-H(7) 0.9300 C(8)-C(1") 1.519(3)

C(8)-C(9) 1.542(3) C(8)-H(8) 0.9800

C(9)-N(10) 1.487(3) C(9)-H(9A) 0.9700

C(9)-H(9B) 0.9700 N(10)-O(12) 1.211(4)

N(10)-O(11) 1.234(4) C(1')-C(2') 1.388(3)

C(1')-C(6') 1.394(3) C(2')-C(3') 1.367(5)

C(2')-C(3B) 1.424(14) C(2')-H(2') 0.9300

C(3')-F(3') 1.350(5) C(3')-C(4') 1.385(5)

C(3B)-C(4') 1.354(14) C(3B)-H(3B) 0.9300

C(4')-C(5') 1.351(5) C(4')-C(5B) 1.398(14)

C(4')-H(4') 0.9300 C(5')-C(6') 1.380(5

C(5')-H(5') 0.9300 C(5B)-F(3B) 1.31(2

C(5B)-C(6') 1.400(14) C(6')-H(6') 0.9300

C(1")-C(6") 1.379(4) C(1")-C(2") 1.381(4)

C(2")-C(3") 1.396(4) C(2")-H(2") 0.9300

C(3")-C(4") 1.372(6) C(3")-H(3") 0.9300

C(4")-C(5") 1.371(6) C(4")-H(4") 0.9300

C(5")-C(6") 1.379(5) C(5")-H(5") 0.9300

C(6")-F(6") 1.360(4)

C(7A)-N(1)-C(2) 109.48(16) C(7A)-N(1)-H(1) 124.7(19)

C(2)-N(1)-H(1) 125.8(19) C(3)-C(2)-N(1) 109.10(16)

C(3)-C(2)-C(1') 131.32(17) N(1)-C(2)-C(1') 119.54(16)

C(2)-C(3)-C(3A) 106.85(16) C(2)-C(3)-C(8) 123.80(17)

C(3A)-C(3)-C(8) 129.17(17) C(7A)-C(3A)-C(4) 117.85(19)

C(7A)-C(3A)-C(3) 106.72(16) C(4)-C(3A)-C(3) 135.42(19)

C(5)-C(4)-C(3A) 119.6(2) C(5)-C(4)-H(4) 120.2

C(3A)-C(4)-H(4) 120.2 C(4)-C(5)-C(6) 119.5(2)

C(4)-C(5)-H(5) 120.3 C(6)-C(5)-H(5) 120.3

C(7)-C(6)-F(6) 117.8(3) C(7)-C(6)-C(5) 124.4(2)

F(6)-C(6)-C(5) 117.8(2) C(6)-C(7)-C(7A) 115.5(2)

C(6)-C(7)-H(7) 122.2 C(7A)-C(7)-H(7) 122.2

N(1)-C(7A)-C(7) 129.1(2) N(1)-C(7A)-C(3A) 107.84(17)

C(7)-C(7A)-C(3A) 123.08(19) C(3)-C(8)-C(1") 115.07(19)

C(3)-C(8)-C(9) 112.87(18) C(1")-C(8)-C(9) 107.50(16) C(3)-C(8)-H(8) 107.0

C(1")-C(8)-H(8) 107.0 C(9)-C(8)-H(8) 107.0

**Table 3**. (continued).

_____________________________________________________

N(10)-C(9)-C(8) 111.12(18) N(10)-C(9)-H(9A) 109.4

C(8)-C(9)-H(9A) 109.4 N(10)-C(9)-H(9B) 109.4

C(8)-C(9)-H(9B) 109.4 H(9A)-C(9)-H(9B) 108.0

O(12)-N(10)-O(11) 124.2(3) O(12)-N(10)-C(9) 118.5(3)

O(11)-N(10)-C(9) 117.3(3) C(2')-C(1')-C(6') 118.72(19)

C(2')-C(1')-C(2) 121.23(17) C(6')-C(1')-C(2) 120.06(19)

C(3')-C(2')-C(1') 119.5(3) C(1')-C(2')-C(3B) 119.4(8)

C(3')-C(2')-H(2') 120.3 C(1')-C(2')-H(2') 120.3

F(3')-C(3')-C(2') 119.0(3) F(3')-C(3')-C(4') 118.5(3)

C(2')-C(3')-C(4') 122.5(4) C(4')-C(3B)-C(2') 120.5(14)

C(4')-C(3B)-H(3B) 119.7 C(2')-C(3B)-H(3B) 119.7

C(5')-C(4')-C(3') 117.2(3) C(3B)-C(4')-C(5B) 121.3(11)

C(5')-C(4')-H(4') 121.4 C(3')-C(4')-H(4') 121.4

C(4')-C(5')-C(6') 122.8(4) C(4')-C(5')-H(5') 118.6

C(6')-C(5')-H(5') 118.6 F(3B)-C(5B)-C(4') 122.6(12)

F(3B)-C(5B)-C(6') 119.4(12) C(4')-C(5B)-C(6') 117.9(14)

C(5')-C(6')-C(1') 119.3(3) C(1')-C(6')-C(5B) 122.1(8)

C(5')-C(6')-H(6') 120.3 C(1')-C(6')-H(6') 120.3

C(6")-C(1")-C(2") 116.4(2) C(6")-C(1")-C(8) 120.0(3)

C(2")-C(1")-C(8) 123.6(2) C(1")-C(2")-C(3") 121.4(3)

C(1")-C(2")-H(2") 119.3 C(3")-C(2")-H(2") 119.3

C(4")-C(3")-C(2") 119.5(4) C(4")-C(3")-H(3") 120.2

C(2")-C(3")-H(3") 120.2 C(5")-C(4")-C(3") 120.7(3)

C(5")-C(4")-H(4") 119.7 C(3")-C(4")-H(4") 119.7

C(4")-C(5")-C(6") 118.2(3) C(4")-C(5")-H(5") 120.9

C(6")-C(5")-H(5") 120.9 F(6")-C(6")-C(5") 117.9(3)

F(6")-C(6")-C(1") 118.4(3) C(5")-C(6")-C(1") 123.7(4)

_____________________________________________________________

**Table 4**. Anisotropic displacement parameters (Å2x 103) for GAT1664. The anisotropic

displacement factor exponent takes the form: -2π2[h2a*2U11 + ... + 2 h k a* b* U12]

______________________________________________________________________________

U11 U22 U33 U23 U13 U12

______________________________________________________________________________

N(1) 74(1) 45(1) 48(1) 6(1) 5(1) -8(1)

C(2) 61(1) 45(1) 45(1) 4(1) 1(1) -3(1)

C(3) 66(1) 44(1) 43(1) 6(1) 2(1) -1(1)

C(3A) 62(1) 49(1) 45(1) 6(1) 1(1) 4(1)

C(4) 82(2) 67(1) 45(1) 9(1) 5(1) 8(1)

C(5) 91(2) 87(2) 44(1) -2(1) 1(1) 23(2)

C(6) 85(2) 72(1) 60(1) -20(1) -7(1) 17(1)

F(6) 144(2) 90(1) 81(1) -40(1) -9(1) 19(1)

C(7) 73(1) 51(1) 69(1) -7(1) -2(1) 3(1)

C(7A) 60(1) 48(1) 50(1) 1(1) 0(1) 2(1)

C(8) 80(1) 42(1) 49(1) 9(1) 2(1) -4(1)

C(9) 77(1) 50(1) 77(1) 19(1) -2(1) -5(1)

N(10) 77(2) 55(1) 130(2) 21(1) -16(2) -10(1)

O(11) 96(2) 83(2) 217(3) 20(2) 25(2) 17(1)

O(12) 142(2) 88(1) 138(2) 41(1) -62(2) -11(2)

C(1') 57(1) 59(1) 43(1) 3(1) 4(1) -9(1)

C(2') 77(1) 69(1) 51(1) -4(1) 3(1) 3(1)

C(3') 74(7) 101(2) 59(1) -21(1) 9(1) 2(4)

F(3') 142(2) 128(2) 86(1) -46(1) 6(1) 38(2)

C(3B) 80(40) 106(7) 52(2) -14(2) 5(2) -16(15)

C(4') 75(1) 119(2) 49(1) -7(1) 7(1) -16(1)

C(5') 83(8) 106(2) 47(1) 15(1) 4(1) -16(3)

C(5B) 90(50) 117(8) 46(3) 3(2) 5(3) -13(19)

F(3B) 138(10) 165(8) 64(5) 34(6) 8(5) 15(7)

C(6') 71(1) 72(1) 50(1) 13(1) 2(1) -10(1)

C(1") 82(2) 48(1) 60(1) 13(1) 16(1) 7(1)

C(2") 80(2) 74(1) 67(1) 14(1) 10(1) 16(1)

C(3") 92(2) 108(2) 90(2) 30(2) 16(2) 37(2)

C(4") 127(3) 102(3) 122(3) 44(2) 36(3) 61(2)

C(5") 163(4) 60(2) 130(3) 14(2) 31(3) 38(2)

C(6") 124(2) 53(1) 91(2) 8(1) 15(2) 8(2)

F(6") 198(3) 57(1) 125(2) -13(1) -25(2) -4(1)

______________________________________________________________________________**Table 5**. Hydrogen coordinates (x 104) and isotropic displacement parameters (Å2x 103) for GAT1664.

________________________________________________________________________________

x y z U(eq)

________________________________________________________________________________

H(1) 7270(50) 7870(20) 5170(14) 84

H(4) 4986 5679 7029 78

H(5) 5297 7179 7671 89

H(7) 7186 9025 6261 77

H(8) 5151 4352 5233 68

H(9A) 2905 3622 5878 82

H(9B) 3445 4422 6418 82

H(2') 7927 4467 4716 79

H(3B) 8273 4007 3662 94

H(4') 7427 5208 2909 97

H(5') 6360 6900 3177 94

H(6') 5949 7400 4194 77

H(2") 7920 4617 6572 88

H(3") 9772 3257 6997 116

H(4") 9558 1495 6630 141

H(5") 7593 1089 5815 141

________________________________________________________________________________**Table 6**. Torsion angles [°] for GAT1664.

________________________________________________________________

C(7A)-N(1)-C(2)-C(3) 1.0(3) C(7A)-N(1)-C(2)-C(1') 179.06(19)

N(1)-C(2)-C(3)-C(3A) -0.6(2) C(1')-C(2)-C(3)-C(3A) -178.4(2)

N(1)-C(2)-C(3)-C(8) 174.9(2) C(1')-C(2)-C(3)-C(8) -2.9(4)

C(2)-C(3)-C(3A)-C(7A) 0.1(2) C(8)-C(3)-C(3A)-C(7A) -175.1(2)

C(2)-C(3)-C(3A)-C(4) -179.1(2) C(8)-C(3)-C(3A)-C(4) 5.7(4)

C(7A)-C(3A)-C(4)-C(5) -0.9(3) C(3)-C(3A)-C(4)-C(5) 178.2(2)

C(3A)-C(4)-C(5)-C(6) -0.3(4) C(4)-C(5)-C(6)-C(7) 1.1(4)

C(4)-C(5)-C(6)-F(6) -179.1(2) F(6)-C(6)-C(7)-C(7A) 179.6(2)

C(5)-C(6)-C(7)-C(7A) -0.6(4) C(2)-N(1)-C(7A)-C(7) 177.5(2)

C(2)-N(1)-C(7A)-C(3A) -0.9(2) C(6)-C(7)-C(7A)-N(1) -178.8(2)

C(6)-C(7)-C(7A)-C(3A) -0.6(4) C(4)-C(3A)-C(7A)-N(1) 179.9(2)

C(3)-C(3A)-C(7A)-N(1) 0.5(2) C(4)-C(3A)-C(7A)-C(7) 1.4(3)

C(3)-C(3A)-C(7A)-C(7) -178.0(2) C(2)-C(3)-C(8)-C(1") 107.2(2)

C(3A)-C(3)-C(8)-C(1") -78.4(3) C(2)-C(3)-C(8)-C(9) -129.0(2)

C(3A)-C(3)-C(8)-C(9) 45.5(3) C(3)-C(8)-C(9)-N(10) 50.8(3)

C(1")-C(8)-C(9)-N(10) 178.7(2) C(8)-C(9)-N(10)-O(12) 45.7(3)

C(8)-C(9)-N(10)-O(11) -132.3(3) C(3)-C(2)-C(1')-C(2') -39.4(4)

N(1)-C(2)-C(1')-C(2') 143.0(2) C(3)-C(2)-C(1')-C(6') 140.0(2)

N(1)-C(2)-C(1')-C(6') -37.6(3) C(6')-C(1')-C(2')-C(3') -2.2(4)

C(2)-C(1')-C(2')-C(3') 177.3(3) C(6')-C(1')-C(2')-C(3B) -0.9(2)

C(2)-C(1')-C(2')-C(3B) 178.6(3) C(1')-C(2')-C(3')-F(3') -177.7(3)

C(1')-C(2')-C(3')-C(4') 2.0(6) C(1')-C(2')-C(3B)-C(4') -0.6(4)

C(2')-C(3B)-C(4')-C(5B) 1.3(8) F(3')-C(3')-C(4')-C(5') 179.3(3)

C(2')-C(3')-C(4')-C(5') -0.4(6) C(3')-C(4')-C(5')-C(6') -1.1(6)

C(3B)-C(4')-C(5B)-F(3B) -176.4(10) C(3B)-C(4')-C(5B)-C(6') -0.5(12)

C(4')-C(5')-C(6')-C(1') 0.9(5) C(2')-C(1')-C(6')-C(5') 0.8(3)

C(2)-C(1')-C(6')-C(5') -178.7(3) C(2')-C(1')-C(6')-C(5B) 1.7(6)

C(2)-C(1')-C(6')-C(5B) -177.7(6) F(3B)-C(5B)-C(6')-C(1') 175.0(10)

C(4')-C(5B)-C(6')-C(1') -1.1(11) C(3)-C(8)-C(1")-C(6") -153.4(2)

C(9)-C(8)-C(1")-C(6") 80.0(3) C(3)-C(8)-C(1")-C(2") 26.5(3)

C(9)-C(8)-C(1")-C(2") -100.2(3) C(6")-C(1")-C(2")-C(3") -2.6(4)

C(8)-C(1")-C(2")-C(3") 177.6(2) C(1")-C(2")-C(3")-C(4") 0.7(5)

C(2")-C(3")-C(4")-C(5") 1.5(5) C(3")-C(4")-C(5")-C(6") -1.5(6)

C(4")-C(5")-C(6")-F(6") -180.0(3) C(4")-C(5")-C(6")-C(1") -0.5(6)

C(2")-C(1")-C(6")-F(6") -178.0(3) C(8)-C(1")-C(6")-F(6") 1.9(4)

C(2")-C(1")-C(6")-C(5") 2.5(5) C(8)-C(1")-C(6")-C(5") -177.6(3)

________________________________________________________________

**Table 7**. Hydrogen bonds for GAT1664 [Å and °].

____________________________________________________________________________

D-H...A d(D-H) d(H...A) d(D...A) <(DHA)

____________________________________________________________________________

N(1)-H(1)...O(12)#1 0.86(3) 2.33(3) 3.102(3) 150(3)

C(5)-H(5)...F(3')#2 0.93 2.54 3.284(3) 137.4

C(9)-H(9B)...F(6)#3 0.97 2.57 3.511(3) 165.0

C(6')-H(6')...O(11)#1 0.93 2.48 3.280(4) 143.6

____________________________________________________________________________

Symmetry transformations used to generate equivalent atoms:

#1 x+1/2,-y+3/2,-z+1 #2 -x+3/2,-y+1,z+1/2 #3 -x+1,y-1/2,-z+3/2

**Single-crystal X-ray Diffraction Analysis of GAT1666.**

C_22_H_15_F_3_N_2_O_2_, FW = 396.36, tetragonal, P4_3_, a = 7.42160(7) Å, b = 7.42160(7) Å, c = 34.2998(5) Å, *α* = 90°, *β* = 90°, *γ* = 90°, V = 1889.24(4) Å^3^, Z = 4, *ρ*calc (293K) = 1.394 Mg/m^3^, *μ* = 0.939 mm^-1^, *F*(000) = 816, R_1_ = 0.0271 for 3750 observed (I > 2σI) reflections and 0.0275 for all 3799 reflections, Goodness-of-fit = 1.053, 265 parameters.

A clear colorless plate crystal of dimensions 0.402 x 0.256 x 0.050 mm was mounted on a MiteGen MicroMesh using a small amount of Cargille Immersion Oil. Data were collected on a Bruker three-circle platform diffractometer equipped with a PHOTON II CPAD detector. The crystals were irradiated using a 1μs microfocus CuK_α_ source (λ = 1.54178) with Montel optics. Data was collected at room temperature (20°C).

Data collection was performed and the unit cell was initially refined using *APEX3* [v2015.5-2].^1^ Data Reduction was performed using *SAINT* [v8.34A]^2^ and *XPREP* [v2014/2]^3^. Corrections were applied for Lorentz, polarization, and absorption effects using *SADABS* [v2014/2].^4^ The structure was solved and refined with the aid of the program SHELXL-2014/7.^5^ The full-matrix least-squares refinement on F^2^ included atomic coordinates and anisotropic thermal parameters for all non-H atoms. Hydrogen atoms were located from the difference electron-density maps and added using a riding model.

**Table 8**. Crystal data and structure refinement for GAT1666.

Identification code gat1666

Empirical formula C_22_H_15_F_3_N_2_O_2_

Formula weight 396.36

Temperature 293(2) K

Wavelength 1.54178 Å

Crystal system Tetragonal

Space group P4_3_

Unit cell dimensions a = 7.42160(7) Å α= 90°.

b = 7.42160(7) Å β= 90°.

c = 34.2998(5) Å γ = 90°.

Volume 1889.24(4) Å3

Z 4

Density (20°C) 1.394 Mg/m3

Absorption coefficient 0.939 mm-1

F(000) 816

Crystal size 0.402 x 0.256 x 0.050 mm3

Theta range for data collection 6.499 to 74.411°.

Index ranges -9<=h<=9, -8<=k<=8, -40<=l<=42

Reflections collected 27445

Independent reflections 3799 [R_int_ = 0.0254]

Completeness to theta = 67.679° 99.1 %

Absorption correction Semi-empirical from equivalents

Max. and min. transmission 0.7538 and 0.6734

Refinement method Full-matrix least-squares on F2

Data / restraints / parameters 3799 / 1 / 265

Goodness-of-fit on F2 1.053

Final R indices [I>2sigma(I)] R_1_ = 0.0271, wR_2_ = 0.0727

R indices (all data) R_1_ = 0.0275, wR_2_ = 0.0730

Absolute structure parameter 0.04(2)

Largest diff. peak and hole 0.089 and -0.117 e.Å-3

**Table 9**. Atomic coordinates (x 104) and equivalent isotropic displacement parameters (Å2x 103)

for GAT1666. U(eq) is defined as one third of the trace of the orthogonalized Uij tensor.

________________________________________________________________________________

x y z U(eq)

________________________________________________________________________________

N(1) 3050(2) 6918(2) 4522(1) 43(1)

C(2) 3927(2) 7471(2) 4856(1) 39(1)

C(3) 5442(2) 6436(2) 4910(1) 39(1)

C(3A) 5509(2) 5167(2) 4592(1) 42(1)

C(4) 6690(3) 3779(3) 4477(1) 53(1)

C(5) 6281(4) 2793(3) 4149(1) 66(1)

C(6) 4742(4) 3108(3) 3930(1) 70(1)

C(7) 3593(3) 4456(3) 4039(1) 56(1)

F(7) 2067(2) 4813(2) 3836(1) 79(1)

C(7A) 3971(3) 5503(2) 4363(1) 44(1)

C(8) 6833(2) 6768(2) 5222(1) 41(1)

C(9) 8685(2) 7138(2) 5039(1) 48(1)

N(10) 8596(2) 8732(2) 4776(1) 52(1)

O(11) 7824(3) 10058(2) 4890(1) 91(1)

O(12) 9346(2) 8627(3) 4458(1) 70(1)

C(1') 7037(2) 5291(3) 5527(1) 44(1)

C(2') 6360(3) 3555(3) 5484(1) 54(1)

C(3') 6588(4) 2273(3) 5774(1) 68(1)

C(4') 7485(3) 2679(4) 6112(1) 69(1)

C(5') 8176(3) 4383(4) 6163(1) 66(1)

C(6') 7941(2) 5635(3) 5873(1) 52(1)

F(6') 8619(2) 7323(2) 5927(1) 79(1)

C(1") 3184(2) 8968(2) 5087(1) 40(1)

C(2") 2641(2) 10566(3) 4905(1) 50(1)

C(3") 1847(3) 11884(3) 5130(1) 62(1)

F(3") 1302(2) 13410(2) 4950(1) 98(1)

C(4") 1580(3) 11712(3) 5523(1) 70(1)

C(5") 2133(3) 10138(4) 5702(1) 69(1)

C(6") 2937(3) 8781(3) 5486(1) 53(1)

________________________________________________________________________________**Table 10**. Bond lengths [Å] and angles [°] for GAT1666.

_____________________________________________________

N(1)-C(7A) 1.366(2) N(1)-C(2) 1.380(2)

N(1)-H(1) 0.81(3) C(2)-C(3) 1.375(2)

C(2)-C(1") 1.471(2) C(3)-C(3A) 1.443(2)

C(3)-C(8) 1.505(2) C(3A)-C(7A) 1.408(2)

C(3A)-C(4) 1.409(3) C(4)-C(5) 1.377(3)

C(4)-H(4) 0.9300 C(5)-C(6) 1.386(4)

C(5)-H(5) 0.9300 C(6)-C(7) 1.367(3)

C(6)-H(6) 0.9300 C(7)-F(7) 1.355(3)

C(7)-C(7A) 1.385(3) C(8)-C(1') 1.523(2)

C(8)-C(9) 1.536(2) C(8)-H(8) 0.9800

C(9)-N(10) 1.488(2) C(9)-H(9A) 0.9700

C(9)-H(9B) 0.9700 N(10)-O(11) 1.205(2)

N(10)-O(12) 1.227(3) C(1')-C(6') 1.386(3)

C(1')-C(2') 1.391(3) C(2')-C(3') 1.387(3)

C(2')-H(2') 0.9300 C(3')-C(4') 1.369(4)

C(3')-H(3') 0.9300 C(4')-C(5') 1.376(4)

C(4')-H(4') 0.9300 C(5')-C(6') 1.373(3)

C(5')-H(5') 0.9300 C(6')-F(6') 1.363(3)

C(1")-C(6") 1.390(3) C(1")-C(2") 1.399(3)

C(2")-C(3") 1.378(3) C(2")-H(2") 0.9300

C(3")-F(3") 1.352(3) C(3")-C(4") 1.369(4)

C(4")-C(5") 1.382(4) C(4")-H(4") 0.9300

C(5")-C(6") 1.385(3) C(5")-H(5") 0.930

C(6")-H(6") 0.9300

C(7A)-N(1)-C(2) 108.86(14) C(7A)-N(1)-H(1) 127(2)

C(2)-N(1)-H(1) 124(2) C(3)-C(2)-N(1) 109.44(14)

C(3)-C(2)-C(1") 130.90(15) N(1)-C(2)-C(1") 119.66(14)

C(2)-C(3)-C(3A) 106.84(14) C(2)-C(3)-C(8) 124.48(15)

C(3A)-C(3)-C(8) 128.32(14) C(7A)-C(3A)-C(4) 118.49(17)

C(7A)-C(3A)-C(3) 106.19(15) C(4)-C(3A)-C(3) 135.32(17)

C(5)-C(4)-C(3A) 118.73(19) C(5)-C(4)-H(4) 120.6

C(3A)-C(4)-H(4) 120.6 C(4)-C(5)-C(6) 122.3(2)

C(4)-C(5)-H(5) 118.8 C(6)-C(5)-H(5) 118.

C(7)-C(6)-C(5) 119.3(2) C(7)-C(6)-H(6) 120.3

C(5)-C(6)-H(6) 120.3 F(7)-C(7)-C(6) 121.61(19

F(7)-C(7)-C(7A) 118.19(19) C(6)-C(7)-C(7A) 120.2(2)

N(1)-C(7A)-C(7) 130.48(18) N(1)-C(7A)-C(3A) 108.64(16)

C(7)-C(7A)-C(3A) 120.88(17) C(3)-C(8)-C(1') 115.94(14)

C(3)-C(8)-C(9) 110.67(15) C(1')-C(8)-C(9) 108.70(13

C(3)-C(8)-H(8) 107.0 C(1')-C(8)-H(8) 107.0

C(9)-C(8)-H(8) 107.0 N(10)-C(9)-C(8) 110.46(14)

N(10)-C(9)-H(9A) 109.6 C(8)-C(9)-H(9A) 109.6

N(10)-C(9)-H(9B) 109.6 C(8)-C(9)-H(9B) 109.6

**Table 10**. (continued).

_____________________________________________________

H(9A)-C(9)-H(9B) 108.1 O(11)-N(10)-O(12) 123.8(2)

O(11)-N(10)-C(9) 118.3(2) O(12)-N(10)-C(9) 117.87(17

C(6')-C(1')-C(2') 115.85(18) C(6')-C(1')-C(8) 120.25(17)

C(2')-C(1')-C(8) 123.90(16) C(3')-C(2')-C(1') 121.0(2)

C(3')-C(2')-H(2') 119.5 C(1')-C(2')-H(2') 119.5

C(4')-C(3')-C(2') 121.0(2) C(4')-C(3')-H(3') 119.

C(2')-C(3')-H(3') 119.5 C(3')-C(4')-C(5') 119.4(2)

C(3')-C(4')-H(4') 120.3 C(5')-C(4')-H(4') 120.

C(6')-C(5')-C(4') 118.8(2) C(6')-C(5')-H(5') 120.6

C(4')-C(5')-H(5') 120.6 F(6')-C(6')-C(5') 118.37(19)

F(6')-C(6')-C(1') 117.74(19) C(5')-C(6')-C(1') 123.9(2)

C(6")-C(1")-C(2") 119.03(17) C(6")-C(1")-C(2) 120.33(16)

C(2")-C(1")-C(2) 120.57(17) C(3")-C(2")-C(1") 118.4(2)

C(3")-C(2")-H(2") 120.8 C(1")-C(2")-H(2") 120.8

F(3")-C(3")-C(4") 119.0(2) F(3")-C(3")-C(2") 117.9(2

C(4")-C(3")-C(2") 123.1(2) C(3")-C(4")-C(5") 118.30(19)

C(3")-C(4")-H(4") 120.8 C(5")-C(4")-H(4") 120.8

C(4")-C(5")-C(6") 120.3(2) C(4")-C(5")-H(5") 119.9

C(6")-C(5")-H(5") 119.9 C(5")-C(6")-C(1") 120.8(2)

C(5")-C(6")-H(6") 119.6 C(1")-C(6")-H(6") 119.6

_____________________________________________________________

**Table 11**. Anisotropic displacement parameters (Å2x 103) for GAT1666. The anisotropic

displacement factor exponent takes the form: -2π2[h2a*2U11 + ... + 2 h k a* b* U12]

______________________________________________________________________________

U11 U22 U33 U23 U13 U12

______________________________________________________________________________

N(1) 39(1) 50(1) 41(1) -1(1) -9(1) 5(1)

C(2) 34(1) 42(1) 40(1) -1(1) -4(1) -1(1)

C(3) 36(1) 37(1) 43(1) -1(1) -3(1) 1(1)

C(3A) 44(1) 39(1) 42(1) -1(1) 0(1) 0(1)

C(4) 59(1) 45(1) 56(1) -3(1) -2(1) 10(1)

C(5) 88(2) 49(1) 61(1) -13(1) 4(1) 16(1)

C(6) 104(2) 57(1) 49(1) -14(1) -9(1) 6(1)

C(7) 72(1) 56(1) 41(1) -4(1) -11(1) 0(1)

F(7) 91(1) 87(1) 59(1) -16(1) -34(1) 6(1)

C(7A) 49(1) 43(1) 39(1) 0(1) -2(1) 0(1)

C(8) 34(1) 41(1) 48(1) -3(1) -7(1) 2(1)

C(9) 35(1) 47(1) 62(1) 6(1) -6(1) 2(1)

N(10) 41(1) 48(1) 68(1) 7(1) -7(1) -1(1)

O(11) 93(1) 48(1) 131(2) 16(1) 25(1) 15(1)

O(12) 60(1) 92(1) 58(1) 16(1) -9(1) 0(1)

C(1') 34(1) 53(1) 43(1) -1(1) -3(1) 4(1)

C(2') 56(1) 56(1) 50(1) 3(1) -7(1) -6(1)

C(3') 76(2) 64(1) 65(1) 14(1) 2(1) -2(1)

C(4') 69(1) 84(2) 54(1) 20(1) 2(1) 14(1)

C(5') 53(1) 105(2) 42(1) 1(1) -6(1) 12(1)

C(6') 41(1) 67(1) 48(1) -7(1) -5(1) 1(1)

F(6') 83(1) 87(1) 67(1) -18(1) -21(1) -20(1)

C(1") 28(1) 47(1) 46(1) -4(1) -3(1) 2(1)

C(2") 41(1) 50(1) 59(1) 2(1) 2(1) 6(1)

C(3") 45(1) 47(1) 94(2) -5(1) 3(1) 9(1)

F(3") 94(1) 58(1) 141(2) 13(1) 17(1) 29(1)

C(4") 54(1) 70(1) 86(2) -30(1) 10(1) 9(1)

C(5") 64(1) 89(2) 55(1) -22(1) 4(1) 7(1)

C(6") 49(1) 62(1) 46(1) -5(1) -3(1) 4(1)

______________________________________________________________________________**Table 12**. Hydrogen coordinates ( x 104) and isotropic displacement parameters (Å2x 103) for GAT1666.

________________________________________________________________________________

x y z U(eq)

________________________________________________________________________________

H(1) 2080(40) 7300(40) 4452(9) 65

H(4) 7727 3534 4620 64

H(5) 7064 1883 4071 79

H(6) 4494 2408 3711 84

H(8) 6474 7867 5360 49

H(9A) 9562 7355 5243 58

H(9B) 9072 6091 4891 58

H(2') 5746 3248 5257 65

H(3') 6124 1121 5739 82

H(4') 7627 1810 6304 83

H(5') 8790 4681 6390 80

H(2") 2811 10735 4639 60

H(4") 1041 12630 5666 84

H(5") 1965 9989 5969 83

H(6") 3316 7733 5610 63

________________________________________________________________________________**Table 13**. Torsion angles [°] for GAT1666.

________________________________________________________________

C(7A)-N(1)-C(2)-C(3) 1.2(2) C(7A)-N(1)-C(2)-C(1") -179.02(15)

N(1)-C(2)-C(3)-C(3A) -0.03(19) C(1")-C(2)-C(3)-C(3A) -179.82(17)

N(1)-C(2)-C(3)-C(8) 173.54(16) C(1")-C(2)-C(3)-C(8) -6.3(3)

C(2)-C(3)-C(3A)-C(7A) -1.06(19) C(8)-C(3)-C(3A)-C(7A) -174.31(17)

C(2)-C(3)-C(3A)-C(4) 179.4(2) C(8)-C(3)-C(3A)-C(4) 6.2(3)

C(7A)-C(3A)-C(4)-C(5) -0.9(3) C(3)-C(3A)-C(4)-C(5) 178.6(2)

C(3A)-C(4)-C(5)-C(6) -0.6(4) C(4)-C(5)-C(6)-C(7) 0.8(4)

C(5)-C(6)-C(7)-F(7) -179.7(2) C(5)-C(6)-C(7)-C(7A) 0.4(4)

C(2)-N(1)-C(7A)-C(7) 177.32(19) C(2)-N(1)-C(7A)-C(3A) -1.9(2)

F(7)-C(7)-C(7A)-N(1) -0.9(3) C(6)-C(7)-C(7A)-N(1) 179.0(2)

F(7)-C(7)-C(7A)-C(3A) 178.23(19) C(6)-C(7)-C(7A)-C(3A) -1.9(3)

C(4)-C(3A)-C(7A)-N(1) -178.59(17) C(3)-C(3A)-C(7A)-N(1) 1.8(2)

C(4)-C(3A)-C(7A)-C(7) 2.1(3) C(3)-C(3A)-C(7A)-C(7) -177.48(17)

C(2)-C(3)-C(8)-C(1') 116.38(18) C(3A)-C(3)-C(8)-C(1') -71.5(2)

C(2)-C(3)-C(8)-C(9) -119.24(18) C(3A)-C(3)-C(8)-C(9) 52.9(2)

C(3)-C(8)-C(9)-N(10) 58.94(19) C(1')-C(8)-C(9)-N(10) -172.65(15)

C(8)-C(9)-N(10)-O(11) 45.5(2) C(8)-C(9)-N(10)-O(12) -135.90(18)

C(3)-C(8)-C(1')-C(6') -164.47(16) C(9)-C(8)-C(1')-C(6') 70.1(2)

C(3)-C(8)-C(1')-C(2') 15.6(2) C(9)-C(8)-C(1')-C(2') -109.8(2)

C(6')-C(1')-C(2')-C(3') 0.3(3) C(8)-C(1')-C(2')-C(3') -179.8(2)

C(1')-C(2')-C(3')-C(4') 0.0(4) C(2')-C(3')-C(4')-C(5') -0.2(4)

C(3')-C(4')-C(5')-C(6') 0.1(3) C(4')-C(5')-C(6')-F(6') 179.5(2)

C(4')-C(5')-C(6')-C(1') 0.3(3) C(2')-C(1')-C(6')-F(6') -179.71(18)

C(8)-C(1')-C(6')-F(6') 0.4(3) C(2')-C(1')-C(6')-C(5') -0.4(3)

C(8)-C(1')-C(6')-C(5') 179.66(18) C(3)-C(2)-C(1")-C(6") -50.7(3)

N(1)-C(2)-C(1")-C(6") 129.50(18) C(3)-C(2)-C(1")-C(2") 132.2(2)

N(1)-C(2)-C(1")-C(2") -47.6(2) C(6")-C(1")-C(2")-C(3") -1.2(3)

C(2)-C(1")-C(2")-C(3") 175.89(17) C(1")-C(2")-C(3")-F(3") -178.93(18)

C(1")-C(2")-C(3")-C(4") 0.6(3) F(3")-C(3")-C(4")-C(5") 179.5(2)

C(2")-C(3")-C(4")-C(5") -0.1(4) C(3")-C(4")-C(5")-C(6") 0.1(4)

C(4")-C(5")-C(6")-C(1") -0.7(4) C(2")-C(1")-C(6")-C(5") 1.3(3)

C(2)-C(1")-C(6")-C(5") -175.85(18) ________________________________________________________________

**Table 14**. Hydrogen bonds for GAT1666 [Å and °].

____________________________________________________________________________

D-H...A d(D-H) d(H...A) d(D...A) <(DHA)

____________________________________________________________________________

N(1)-H(1)...O(12)#1 0.81(3) 2.25(3) 3.035(2) 162(3)

C(9)-H(9A)...F(6') 0.97 2.45 3.052(3) 119.9

C(9)-H(9B)...F(3")#2 0.97 2.60 3.394(2) 139.6

____________________________________________________________________________

Symmetry transformations used to generate equivalent atoms:

#1 x-1,y,z #2 x+1,y-1,z

**References:**

1. Bruker (2015). APEX3 v2015.5-2. Bruker AXS Inc., Madison, Wisconsin, USA.
2. Bruker (2013). SAINT v8.34A. Bruker AXS Inc., Madison, Wisconsin, USA.
3. Bruker (2014). XPREP v2014/2. Bruker AXS Inc., Madison, Wisconsin, USA.
4. Bruker (2014). SADABS v2014/5, Bruker AXS Inc., Madison, Wisconsin, USA.
5. Sheldrick, G. M. (2014). SHELXL-2014/7. University of Göttingen, Germany.
